# Exploring the role of neutrophils in inflammatory pain hypersensitivity via single-cell transcriptome profiling

**DOI:** 10.3389/fimmu.2025.1552993

**Published:** 2025-05-28

**Authors:** Kai Ding, Xing Liu, Bin Zeng, Songyu Liu, Lu Zhang, Bo Li, Jinyi Zhou, Xiaosan Su, Jun Wang

**Affiliations:** ^1^ Department of Anesthesiology, The First Affiliated Hospital of Kunming Medical University, Kunming, China; ^2^ Department of Anesthesiology, The Affiliated Yan’an Hospital of Kunming Medical University, Kunming, China; ^3^ School of Basic Medical Sciences, Yunnan University of Chinese Medicine, Kunming, China; ^4^ Department of Neurosurgery, The Third Affiliated Hospital of Kunming Medical University, Kunming, China; ^5^ Scientific Research and Experimental Center, Yunnan University of Chinese Medicine, Kunming, China

**Keywords:** inflammation, pain, neutrophil, CXCR2, NAMO

## Abstract

**Introduction:**

Myeloid CD11b^+^ cells are crucial mediators in post-operative and CFA-induced inflammation, but their role in pain, particularly the role of neutrophils, is still debated. This study employs single-cell RNA sequencing (scRNA-seq) to analyze CD11b^+^ cell composition in mice after surgery and CFA treatment and investigates the effects and mechanisms of Nicotinamide N-oxide (NAMO) on neutrophils and pain.

**Methods:**

scRNA-seq was used to analyze the transcriptomes of CD11b^+^ cells in murine models of post-operative and CFA-induced inflammation. Using comprehensive bioinformatics techniques, we identified distinct cell subpopulations and characterized their gene expression profiles and functional attributes. Based on these analyses, NAMO was selected to intervene in neutrophil differentiation and maturation. The role of the CXCR2 target gene and NAMO in modulating post-operative and inflammatory pain was then evaluated, exploring potential mechanisms.

**Results:**

scRNA-seq revealed a significant increase in neutrophils and a decrease in monocytes among CD11b^+^ cells following surgery and CFA treatment. Neutrophils comprised seven subpopulations at various differentiation stages from immature to mature. Given the high expression of CXCR2 in neutrophils, we used the CXCR2 inhibitor NAMO to suppress neutrophil differentiation and maturation, which subsequently alleviated post-operative and CFA-induced pain in mice. Proteomics analysis showed that NAMO treatment significantly reduced the expression of S100b and CaMKIIβ proteins in mouse neutrophils.

**Discussion:**

Following surgery and CFA treatment, mature neutrophils were significantly elevated. The CXCR2 antagonist NAMO alleviated post-surgical and CFA-induced pain by inhibiting neutrophil differentiation and maturation. These findings offer novel approaches for pain prevention and treatment.

## 1 Introduction

Inflammatory pain represents a significant public health challenge, adversely impacting quality of life and imposing considerable economic costs (1, 2). This type of pain results from tissue injury followed by an immune response, leading to increased pain sensitivity and chronic pain (3). The mechanisms underlying inflammatory pain are complex and involve intricate interactions between immune cells and their molecular mediators. Several critical processes drive the pathogenesis of inflammatory pain. Immune cells release proinflammatory cytokines and chemokines that sensitize nociceptors, thereby amplifying pain perception (4). Neutrophils play a notable role by releasing reactive oxygen species, proteases, and other signaling molecules that exacerbate inflammation and pain (5, 6). Despite extensive research, a comprehensive understanding of the specific roles of neutrophils and other immune cells in this context remains incomplete and requires further investigation.

In the context of inflammation and tissue injury, both resident and infiltrating leukocytes produce mediators that can either exacerbate or relieve pain. However, the specific roles of different cell populations in modulating pain remain a matter of debate. Ghasemlou et al. identified proliferating CD11b^+^Ly6G^−^ myeloid cells as key contributors to mechanical hypersensitivity in incisional pain and, to a lesser extent, CFA-induced inflammation (7). In contrast, other studies have indicated that neutrophils involved in CXCL1-CXCR1/2 signaling play a significant role in the inflammatory cascade, leading to mechanical hyperalgesia (8). Neutrophil depletion via vinblastine sulfate or anti-neutrophil antibodies alleviated mechanical hyperalgesia following a foot incision. Similarly, the oral CXCR1/2 antagonist ladarixin reduced mechanical hyperalgesia and neutrophil infiltration at the incision site (9). Sahbaie et al. reported that neutrophil depletion via an anti-Gr1 antibody did not alter mechanical hypersensitivity postincision but did slightly increase thermal sensitivity while also reducing paw edema and the levels of interleukin-1 and C5a (10).

Research on T cells indicates that γδ T cells modulate bone marrow cell recruitment in models of peripheral inflammatory pain, such as those induced by formalin injection, incision, and CFA injection, without affecting baseline sensitivity or altering mechanical or thermal hypersensitivity after tissue damage (11). Additionally, antigen-stimulated local opioid release from CD4^+^ T lymphocytes has been shown to alleviate CFA-induced inflammatory pain (12). Mitsui et al. (13) demonstrated that mice deficient in macrophage autophagy presented increased postoperative pain and inflammation. These mice also showed increased secretion of proinflammatory cytokines and greater monocyte/macrophage infiltration at surgical sites. Other studies have identified mast cell degranulation, along with histamine and serotonin release, as contributors to inflammatory processes and postoperative pain (14). Furthermore, research using a surgical wound model has shown that dendritic cells (DCs) produce CCL17 and CCL22 at lesion sites, which activate CCR4 on peripheral sensory neurons to induce inflammatory pain. The absence of DCs abolished the postoperative pain response (15).

Conflicting studies and recent findings underscore significant gaps in our understanding of the mechanisms through which immune cells influence pain sensitivity. To address these gaps, we employed single-cell RNA sequencing (scRNA-seq), a high-throughput method for assessing gene expression and transcriptional networks at the resolution of individual cells (16). This approach was used to examine the heterogeneity and functional diversity of CD11b^+^ cell populations. Through magnetic sorting, we isolated these cells from peripheral blood during early-stage inflammation induced by CFA injection and surgical incision, with the objective of identifying additional pain-related signaling pathways. We employed two widely used mouse models of inflammatory pain: (i) plantar CFA injection and (ii) plantar incision wounds. Our focus on circulating immune cells stemmed from insufficient infiltrating cell numbers in affected tissues and technical limitations in isolating viable tissue-resident populations. Peripheral blood provided adequate CD11b^+^ cells for robust sequencing while capturing systemic immune dynamics relevant to both localized and generalized hyperalgesia.

## 2 Materials and methods

### 2.1 Experimental animals

Male C57BL/6J mice, aged 6–8 weeks and weighing 20 ± 2 g, were obtained from the Animal Experimental Center of Yunnan University, China. These mice were maintained in a specific pathogen-free (SPF) environment with regulated 12-hour light/dark cycles and were provided unrestricted access to food and water. To minimize variability, the mice were housed according to their specific experimental conditions. Postoperative pain was induced via a refined surgical technique: the mice were anesthetized with 1% pentobarbital sodium (50 mg/kg), and a 5-mm longitudinal incision was made on the plantar surface of the right hind paw, extending through the plantaris muscle (7, 17, 18). The incision was closed with a single 6–0 nylon suture. Sham-operated control mice were subjected to anesthesia for the same duration but did not receive an incision. To induce an inflammatory response via complete Freund’s adjuvant (CFA; Sigma Aldrich, St. Louis, USA), 20 µL of CFA was injected subcutaneously into the plantar region of the right hind paw with a 26-gauge microsyringe (7, 19). Sham-treated mice received a comparable subcutaneous injection of sterile 0.9% saline (20 µL) at the same site.

### 2.2 Assessment of thermal and mechanical hypersensitivity

Thermal hypersensitivity was assessed via a Hargreaves apparatus (UGO37570, Ugo Baseline, Comerio, Italy), which measures paw withdrawal latency (PWL) in response to a heat stimulus. The mice were positioned on a glass surface prewarmed to 30°C within transparent plastic chambers measuring 7 × 5 × 5 cm. Following a 15-minute acclimation period, a focused radiant heat source was applied to the hind paw. The halogen lamp intensity was calibrated to elicit a withdrawal response within 10 to 12 s, with a maximum cutoff set at 20 s to prevent tissue damage. Five trials were conducted, each separated by 5–10 minutes, to calculate the mean PWL. Mechanical hypersensitivity was evaluated via an electronic von Frey anesthesiometer (IITC Life Science Inc., Woodland Hills, CA, USA).

### 2.3 Isolation and purification of CD11b+ cells

Peripheral blood from nine mice was obtained via orbital extraction, with adjacent hair trimmed to prevent contamination. The blood was combined with red blood cell (RBC) lysis buffer (BD Biosciences, San Jose, CA, USA) and incubated at low temperature for 8 to 10 minutes to facilitate RBC lysis. The supernatant was then discarded, and the remaining cell suspension was treated with 2 mM EDTA and 0.5% BSA in PBS (pH 7.2). After the sample was passed through a premoistened 30 µm nylon mesh to prevent clogging, it was centrifuged at 300 × g for 10 minutes. The resulting cell pellet was resuspended in 90 µL of buffer per 10^7^ cells and mixed with 10 µL of CD11b MicroBeads UltraPure (Miltenyi Biotec, Germany), followed by incubation at 2–8°C for 10 minutes. The washed pellet was then resuspended in 500 µL of buffer for up to 10^8^ cells. The cells were isolated via an LS column in a MACS Separator. CD11b^+^ cells, which were magnetically labeled, were collected under sterile, cold conditions to ensure maximum purity.

### 2.4 Flow cytometry

Paw tissue was minced and digested in 1 mg/mL collagenase II (Solarbio, Beijing, China) at 37°C for 2 hours. Following digestion, the reaction was halted with DMEM (VivaCell, Shanghai, China). The mixture was then filtered through a 70 μm mesh and resuspended in PBS with 0.5% BSA (Solarbio, Beijing, China). The cells were treated with FcBlock (BD Biosciences, San Jose, CA, USA) on ice for 10 minutes and then incubated with specific antibodies (anti-CD11b-FITC, anti-CD45-APC-Cy7, anti-Ly-6G-APC/PerCP-Cy5.5, anti-CD14-PE, anti-CXCR2-PE, anti-CD11c-APC, anti-CD3-APC, or anti-NK1.1-BV421) for 30 minutes at 4°C in the dark. The cells were subsequently washed and analyzed via a FACS Celesta flow cytometer (BD Biosciences, San Jose, CA, USA), and the data were processed via FlowJo software (TreeStar, USA). Peripheral blood was collected from the tail vein into EDTA tubes, subjected to red cell lysis for 8–10 minutes, blocked, incubated with antibodies, washed, and prepared for analysis.

### 2.5 Single-cell collection, library construction, and sequencing

Mouse cells were sorted into PBS containing 0.05% BSA following the 10× Genomics protocol. To ensure at least 80% viability, as confirmed by trypan blue staining, the cells were processed within two hours. Single-cell transcriptome libraries were indexed via 10× Genomics droplet technology and sequenced via the Illumina HiSeq PE150 platform (Novogene Bioinformatics Technology, Beijing, China).

### 2.6 Processing of scRNA-seq data

To address the intersample variability arising from biological and technical factors, batch correction was implemented via the ScaleData and Harmony functions. These methods effectively minimize variability related to batch effects and mitochondrial gene expression. Dimension reduction involves three key stages: the selection of variable genes, principal component analysis (PCA), and uniform manifold approximation and projection (UMAP). Variable genes identified through FindVariableGenes were analyzed via PCA via the RunPCA function. Unsupervised clustering was performed with the Louvain algorithm in the FindClusters function at resolutions of 0.2 for all control cells and 0.1 for neutrophils, with visualization achieved through UMAP. Neutrophil clusters G0 to G6 represented varying maturation stages, with G4 to G6 denoting the early stages. Differentially expressed genes (DEGs) were identified via FindMarkers or FindAllMarkers on normalized data, excluding genes with an adjusted p value greater than 0.05 (Bonferroni correction). In a specified experiment (**Supplementary Figure 1**), DEGs within neutrophil subpopulations were determined via the Wilcoxon rank-sum test, which applies a twofold change threshold. Lineage differentiation trajectories were inferred via Monocle 2 and pseudotime analysis with the newCellDataSet. Cell–cell communication was evaluated via the CellChat R package, which analyzes ligand–receptor expression to infer intercellular networks. Cells were scored on the basis of gene signatures indicative of specific biological functions, and gene set enrichment analysis (GSEA) was conducted to compute enrichment scores for gene expression, using reference functional gene sets detailed in **Supplementary Table 6**.

### 2.7 NAMO administration

Nicotinamide N-oxide (NAMO) powder (Selleck, Shanghai, China) was dissolved in distilled water to a concentration of 10 mg/mL. The experimental group received an intraperitoneal injection of 0.6 mL (equivalent to 300 mg/kg), while the control group was administered double-distilled water (ddH_2_O). Injections were initiated two hours prior to the incision and continued daily for 14 days following surgery and CFA treatment.

### 2.8 Isolation and purification of Ly6G+ cells

Peripheral blood from the mice was collected, followed by erythrocyte lysis and resuspension of the cell pellet. Ly6G^+^ cells were isolated via either magnetic activated cell sorting (MACS) or fluorescence activated cell sorting (FACS) for subsequent proteome sequencing, Giemsa staining, and Western blot analysis. Purified neutrophils were cytospun at 5.5 × 10³ rpm for 5 minutes, air-dried, stained with Giemsa solution, and imaged via an inverted microscope (Motic, Fujian, China).

### 2.9 Label-free quantitative proteomics

Label-free quantitative proteomics was carried out by Novogene Bioinformatics Technology Co., Ltd. (Beijing, China) to identify differentially expressed proteins in Ly6G^+^ cells treated with NAMO compared with ddH_2_O controls in CFA-treated mice. The workflow included raw data filtration, quality control, protein function annotation through Gene Ontology, Clusters of Orthologous Groups, KEGG, and InterPro, protein expression quantification, differential expression analysis, enrichment analyses for GO, KEGG, and InterPro pathways, and assessment of protein–protein interactions.

### 2.10 H&E and immunohistochemistry staining

The mice were deeply anesthetized and euthanized via transcardial perfusion with 4% paraformaldehyde in 0.1 M phosphate buffer. The hind paw, including the skin and underlying muscle, was excised and postfixed for 1 hour. It was then cryoprotected by immersion in 30% sucrose in 0.1 M phosphate buffer for 48 hours. Serial 14 μm cryostat sections were prepared for histological analysis and stained with hematoxylin and eosin (H&E) following standard protocols. Immunohistochemical (IHC) analysis was performed with S100b rabbit anti-mouse polyclonal IgG (1:1500; Proteintech Group, Inc., Chicago, USA) and horseradish peroxidase-conjugated goat anti-rabbit IgG (1:200; Servicebio, Hubei, China).

### 2.11 Immunofluorescence

The mice were euthanized at various time points relative to the induction of the pain model, and the right hind foot was collected. The samples were fixed in 4% paraformaldehyde, processed through a graded alcohol series, and embedded in paraffin. Longitudinal sections were then dewaxed, rehydrated, treated with 2 mM HCl at 37°C for 1 hour, and rinsed with boric acid buffer. For dual immunofluorescence, the sections were subjected to trypsin digestion, blocked, and incubated with primary antibodies targeting CD11b, Ly6G (BD Biosciences, San Jose, CA, USA), and BrdU (Servicebio, Hubei, China). The sections were subsequently treated with fluorophore-conjugated secondary antibodies. The slides were examined via an Olympus fluorescence microscope.

### 2.12 Protein ChIP assay

The protein concentration in the pedicle-treated tissues was assessed via a protein microarray kit (GSM-INF-1; RayBio, Guangzhou, China) in accordance with the manufacturer’s instructions. The target cytokines were captured and detected via biotin-labeled antibodies and visualized with a streptavidin-Cy3 dye complex. Spot density was quantified via a laser scanner (InnoScan 300 Microarray Scanner, Innopsys, 31–390 Carbonne, France), followed by normalization and subsequent analysis.

### 2.13 Western blot analysis

Peripheral blood was collected from the mice to isolate leukocytes. Mouse paws were homogenized following the removal of the skin and bones, and Ly6G cells were subsequently sorted. Proteins were extracted for analysis via Western blotting. Equal amounts of protein were resolved by SDS–PAGE via gels with concentrations ranging from 7.5% to 12%, transferred onto PVDF membranes (Sigma–Aldrich, St. Louis, USA), and probed with antibodies against β-actin, GAPDH, S100b, CaMKIIβ, and p-CaMKIIβ (Cell Signaling Technology, Danvers, Massachusetts, USA). Detection was conducted using HRP-conjugated secondary antibodies (dilution 1:10,000, Proteintech Group, Inc., Chicago, USA), and chemiluminescence was visualized with a Tanon imaging system (Tanon, China).

### 2.14 Statistical analyses

Behavioral data were analyzed using two-way repeated-measures ANOVA with time as the within-subjects factor and treatment as the between-subjects factor, followed by Bonferroni *post hoc* adjustment for multiple comparisons. Peripheral blood and plantar tissue datasets were evaluated via one-way or two-way ANOVA (as appropriate to experimental design) with Bonferroni correction. All the statistical tests were two-tailed, with significance set at P < 0.05. The results are presented as the means ± standard deviations. Analyses were performed via SPSS version 26.0 (SPSS Inc., Chicago, IL, USA) and GraphPad Prism version 9.5.0 (GraphPad Software Inc., San Diego, CA, USA).

## 3 Results

### 3.1 Single-cell profiling of CD11b^+^ cells in mouse pain models

**Figure 1:**
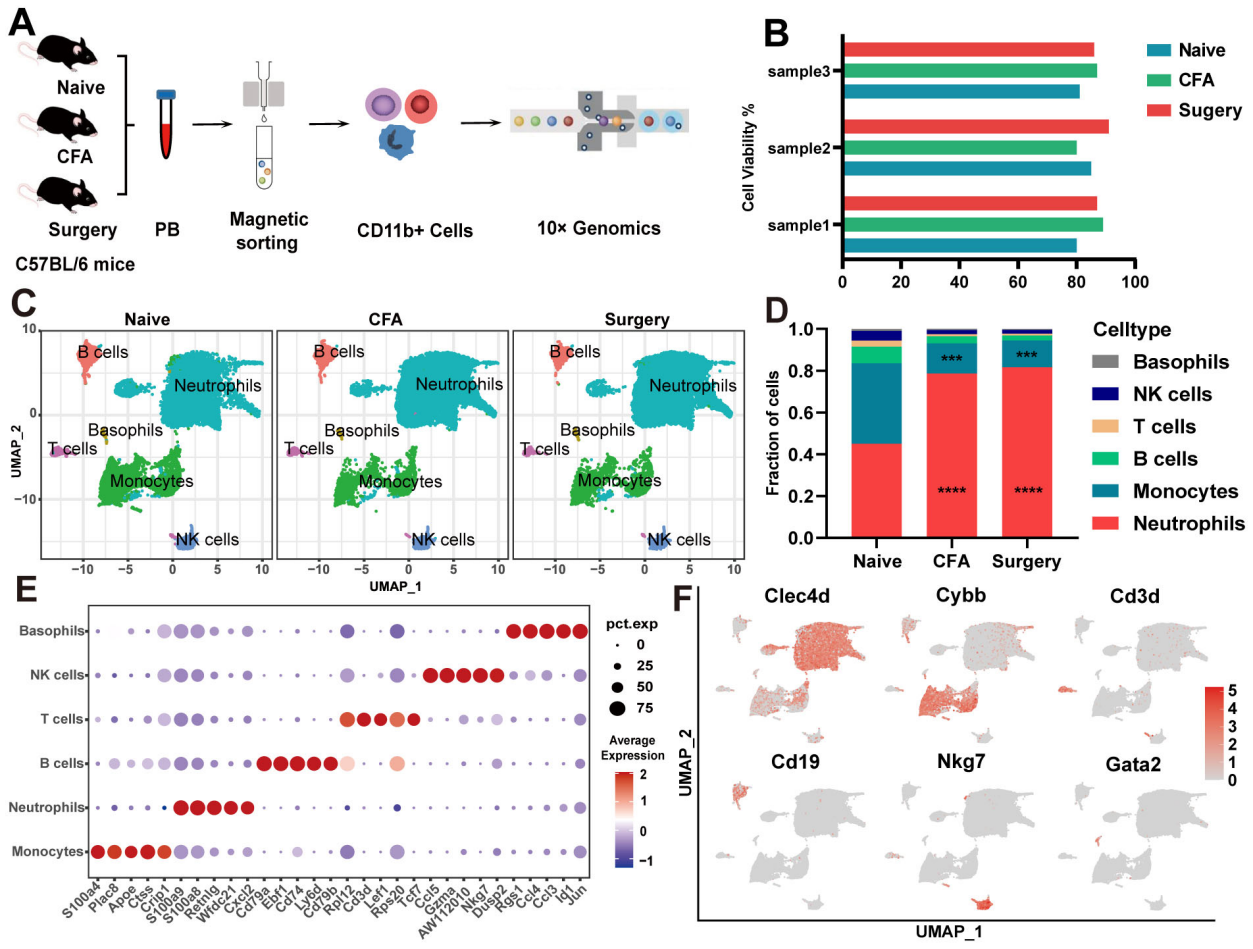
scRNA-seq of CD11b^+^ cells from naïve, surgical, and CFA-treated mice. **(A)** Overview of the single-cell RNA sequencing methodology applied to CD11b^+^ cells. **(B)** Viability percentages of the nine samples evaluated. **(C)** UMAP visualization of CD11b^+^ cells isolated from naïve, surgical, and CFA-treated mice. **(D)** Comparative analysis of the composition of CD11b^+^ cells across the naïve, surgical, and CFA-treated groups. Statistical significance was determined by one-way ANOVA with Bonferroni *post hoc* correction. ***P<0.001, ****P<0.0001 (relative to the naïve group). **(E)** Identification of the top five marker genes for various immune cell types: neutrophils, monocytes, T cells, B cells, NK cells, and basophils. **(F)** Functional annotation highlights of signature genes associated with the aforementioned immune cell subtypes.

Our initial observations revealed significant reductions in mechanical and thermal pain thresholds, particularly within the 6- to 24-hour period following surgical intervention and CFA treatment (**Supplementary Figures 1A–D**). Between 6 hours and 1 day after surgery and CFA treatment, mice exhibited a significant increase in peripheral circulating CD11b^+^ cells, which returned to baseline levels by day 3 (**Supplementary Figures 1E, F**). We subsequently isolated CD11b^+^ cells from naïve, surgery-treated, and CFA-treated mice for scRNA-seq analysis (**Figures 1A, B**). Following quality control measures (**Supplementary Figures 1G–J**, **Supplementary Table 1**), we used the Seurat package to process 50,127 high-quality cells, detecting expression of 16,151 genes. UMAP clustering identified six distinct subsets of CD11b^+^ cells: neutrophils, monocytes, T lymphocytes, B lymphocytes, NK cells, and basophils (**Figure 1C**). Although CD11b^+^ cells predominantly represent cells of myeloid origin, studies have shown that CD11b^+^ cells can express lymphoid markers such as CD3 or B220, particularly when the cells are activated or the organism is in a pathological state (20, 21). Following surgery and CFA treatment, we observed a rapid shift in cell type composition, characterized by a significant increase in neutrophils and a significant decrease in monocytes compared with naïve mice (**Figure 1D**). These subsets were characterized by distinctive gene expression profiles, which were corroborated using the CellMarker and PanglaoDB databases (**Figures 1E, F**, **Supplementary Table 2**). Neutrophils were characterized by upregulation of Clec4d and Cxcl2 expression, while monocytes were characterized by Cybb and S100a4 expression. Other cell types, including T lymphocytes (Cd3d, Lef1), B lymphocytes (Cd19, Cd79a), NK cells (Nkg7, Ccl5), and basophils (Gata2, Ccl4), showed slight decreases in abundance.

Under both treatment conditions, the expression of genes associated with myeloid leukocyte differentiation, migration, and chemotaxis was upregulated in neutrophils (**Supplementary Figures 2A, B**). In CFA-treated mice, neutrophils presented increased gene expression related to leukocyte adhesion and the response to external stimuli, whereas surgery-treated mice presented increased expression of genes associated with oxidative stress and bacterial responses (**Supplementary Figures 2A, B**). Monocytes predominantly expressed genes involved in the negative regulation of the immune response and cytokine production (**Supplementary Figures 2C, D**). CellChat analysis revealed increased interactions between neutrophils and monocytes, alongside reduced interactions with T cells (**Supplementary Figure 2E**). Notable pathways included “CCL” and “FN1”, which are common to both models; “RESISTIN” and “THBS”, which are specific to CFA-treated mice; and “OSM” and “CD96”, which are exclusive to surgery-treated mice (**Supplementary Figure 2F**). Ligand–receptor analysis revealed increased CXCL2-CXCR2 interactions within neutrophils and reduced CCL6-CCR1 interactions between monocytes and neutrophils, as well as increased APP-CD74 interactions with B lymphocytes (**Supplementary Figures 3A, B**). These findings elucidate the intercellular communication networks underlying inflammation and pain in these mouse models.

### 3.2 Single-cell profiling of monocyte differentiation

**Figure 2:**
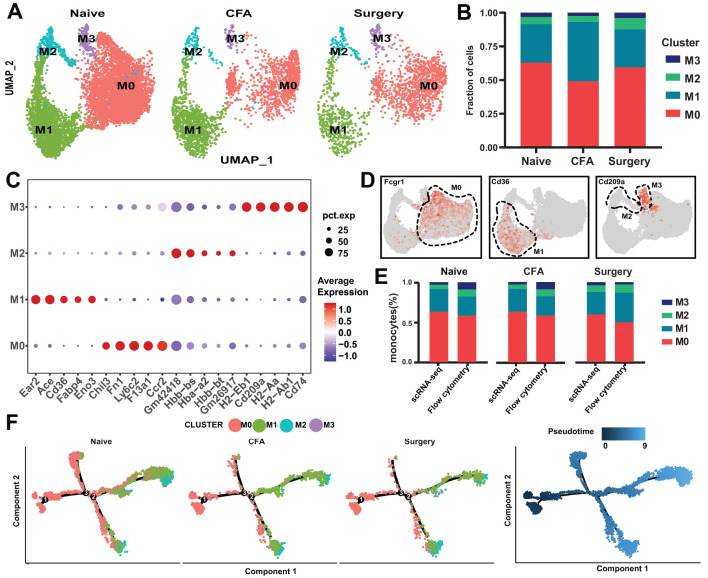
scRNA-seq analysis of monocytes from naïve, surgical, and CFA-treated mice. **(A)** UMAP depiction of monocyte populations from naïve, surgical, and CFA-treated mice. **(B)** Distribution of monocytes across the M0-M3 subsets. **(C)** Expression profiles of the top five marker genes for the M0-M3 subsets. **(D)** Characterization of M0-M3 subsets on the basis of their marker gene expression. **(E)** Comparative analysis of M0-M3 subsets across different models via flow cytometry and single-cell RNA sequencing (percentages derived from panel b; mean ± SD; n = 3–6 mice from two independent experiments). **(F)** Monocle trajectory analysis of monocytes, illustrated by cluster identity (left) and pseudotime sequence (right) as coloring, with single-cell ordering on the basis of variable gene expression.

Using core genes from naïve mice as a reference, we identified four distinct monocyte subsets (M0–M3) in surgery- and CFA-treated mice (**Figures 2A, B**). Each subset demonstrated unique markers, with notable emphasis on Ccr2 and CD14 (**Figure 2C**, **Supplementary Table 3**). Specifically, the M0, M1, and M3 subpopulations were characterized by the expression of Fcgr1, CD36, and CD209a, respectively (**Figure 2D**). Flow cytometry analysis of monocyte subpopulations in the peripheral blood of treated mice confirmed the scRNA-seq findings (**Figure 2E**). Monocle2 pseudotemporal analysis traced the transition of the M0 subset into the M1 and M2 clusters, elucidating distinct functional pathways (**Figure 2F**; **Supplementary Figure 4A**). UMAP visualization depicted M0 as comprising both initial and intermediate states, whereas M1 and M2 represented more advanced states. Gene Ontology (GO) analysis indicated that M0 macrophages were involved in nucleoside triphosphate metabolism and oxidative phosphorylation, whereas M1 macrophages were associated with the regulation of tumor necrosis factor (**Supplementary Figures 4B–E**). CellChat analysis highlighted M0’s critical role in intercellular communication, emphasizing the “APP,” “CCL,” “FN1,” and “CXCL” pathways, with additional engagement in the “THBS,” “CD52,” “OSM,” and “CLEC” pathways posttreatment (**Supplementary Figures 4F, G**). The CLEC pathway, which plays a critical role in immune regulation through pathogen recognition and inflammation modulation (22), was found to be significantly activated in monocyte subsets post-surgery in this study. This activation may promote monocyte-mediated interactions with the tissue microenvironment, thereby driving pain sensitization. The mechanisms underlying this pathway’s role in postoperative pain warrant further in-depth investigation.

### 3.3 Classification and functional analysis of neutrophil subpopulations

**Figure 3:**
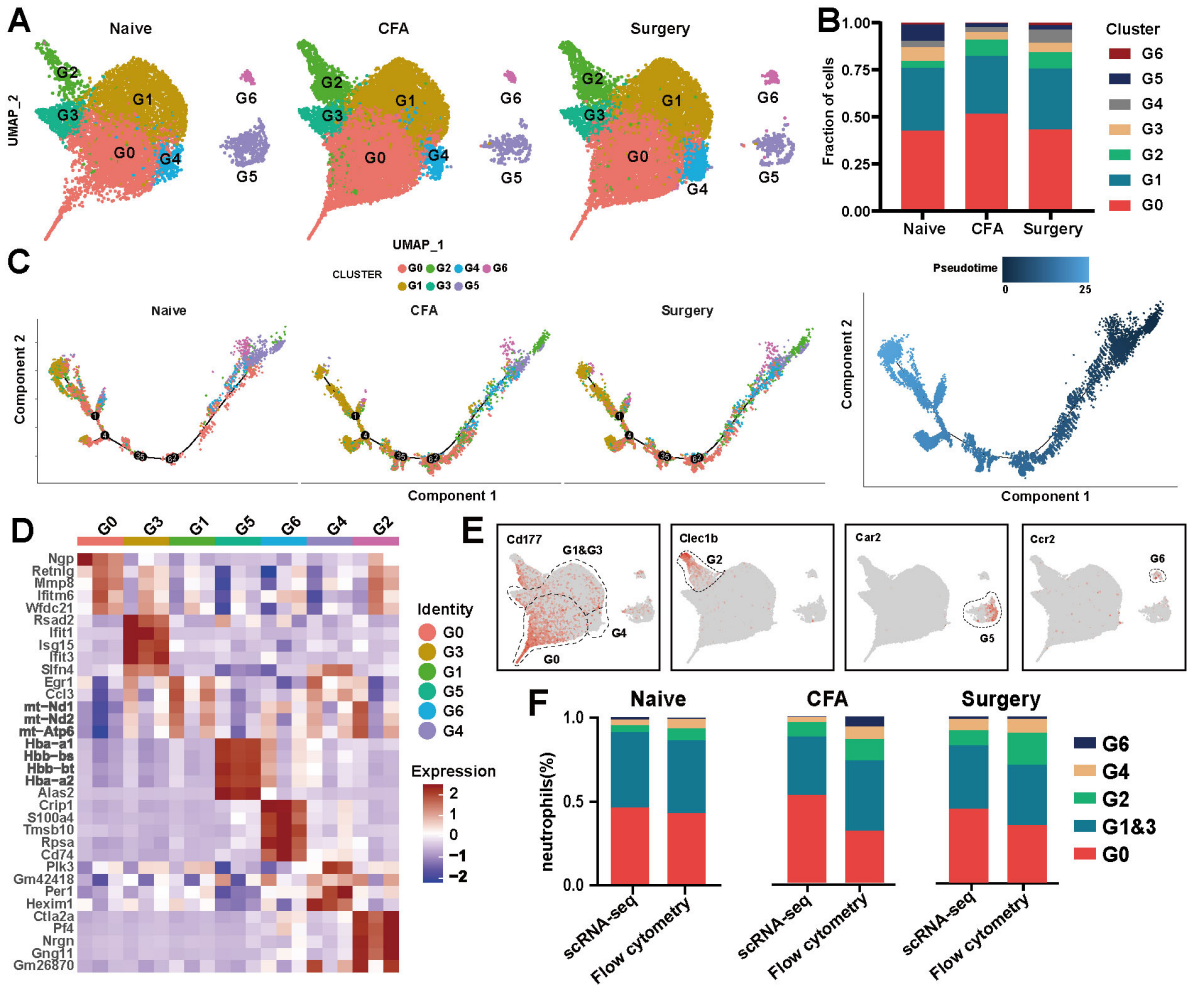
scRNA-seq analysis of neutrophils from naïve, surgical, and CFA-treated mice. **(A)** UMAP visualization of neutrophil subsets in naïve, surgical, and CFA-treated mice. **(B)** Composition analysis of neutrophils from naïve, surgical, and CFA-treated mice. **(C)** Monocle trajectory analysis of neutrophils, with color coding for cluster identity (left) and pseudotime sequence (right). Each dot represents a single cell, ordered by the expression of the most variable genes. **(D)** Heatmap displaying the expression levels of the top five genes defining the G0–G6 subsets. **(E)** Characterization of the G0-G6 subsets on the basis of marker gene expression. **(F)** Comparative analysis of neutrophil subsets in peripheral blood via flow cytometry and single-cell RNA sequencing across the three models.

Neutrophils were categorized into G0–G6 subsets on the basis of their distinctive molecular profiles (**Figure 3A**, **Supplementary Table 4**). Across these subsets, 1,647 differentially expressed genes (DEGs) were identified (**Supplementary Table 5**). The predominant subsets, G0 and G1, demonstrated complex differentiation patterns (**Figure 3B**). Following surgery and CFA treatment, an increased proportion of G2 and a decreased proportion of G3 were observed, indicating a shift toward naïve and intermediate states within G0–G3, whereas G4–G6 retained their respective differentiation stages (**Figures 3B, C**; **Supplementary Figure 5A**). Specifically, G6, G5, and G2 expressed CCR2, Car2, and Clec1b, respectively. G0, G4 and G1&G3 were distinguished by varying levels of CD177 expression (CD177^hi^, CD177^mi^, and CD177^lo^) (**Figures 3D, E**). Flow cytometry analysis corroborated the scRNA-seq data, confirming consistent proportions of G0-G6 cells in the peripheral blood (**Figure 3F**). Notably, the expression of CD14, a monocyte marker, was significantly increased in neutrophils after treatment (**Supplementary Figure 5B**). Both immunofluorescence and flow cytometry confirmed an increase in the number of Ly6G^+^CD14^+^ cells in plantar tissue and peripheral blood after treatment, with the number of Ly6G^+^CD14^+^ cells returning to baseline by days 5–7 (**Supplementary Figures 5C-E**).

### 3.4 Analysis of intercellular communication, cellular function and metabolic activity of neutrophils

**Figure 4:**
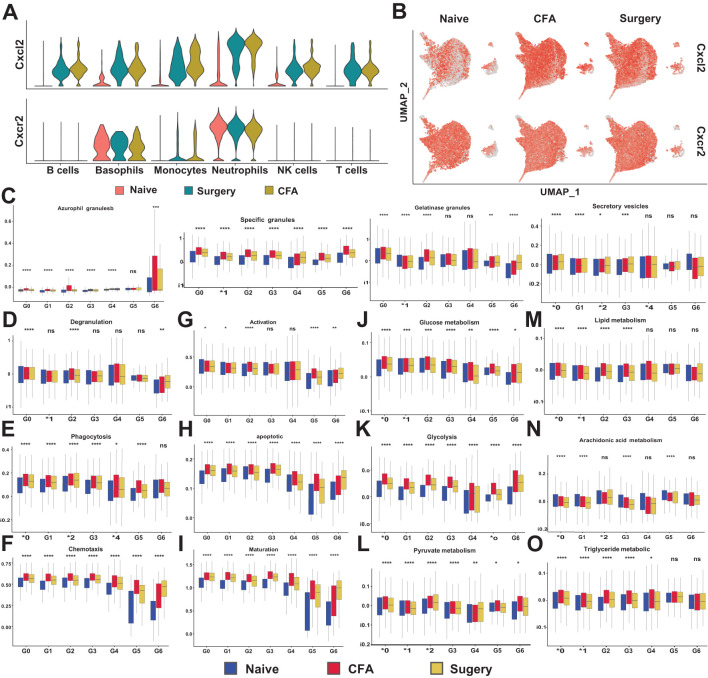
Analysis of CXCL2-CXCR2 expression and functional scores in neutrophils. **(A)** Levels of CXCL2 and CXCR2 enrichment across various immune cell types in naïve, surgical, and CFA-treated mice. **(B)** Expression analysis of CXCL2 and CXCR2 specifically in neutrophils. **(C)** The functional scores of four-level particles of neutrophil in naïve, surgical, and CFA-treated mice. The four granule types are, in sequence: azurophil granules, specific granules, gelatinous granules and secretory vesicles. **(D-I)** Functional scores of neutrophil in naïve, surgical, and CFA-treated mice. Neutrophil degranulation **(D)**, phagocytosis **(E)**, chemotaxis **(F)**, activation **(G)**, apoptosis **(H)** and maturation **(I)**. **(J-O)** Changes in neutrophil metabolism in naïve, surgical, and CFA-treated mice. **(J-L)** Changes in glycometabolism-related functions. Glucose metabolism **(J)**, glycolysis **(K)** and pyruvate metabolism **(L)**. **(M-O)** Scores of lipid metabolism related functions. Lipid metabolism **(M)**, arachidonic acid metabolism **(N)** and triglyceride metabolism **(O)**. *P < 0.05, **P < 0.01, ***P < 0.001, ****P < 0.0001, ns, not statistically.

CellChat analysis revealed distinct communication roles among neutrophil subsets. In CFA-treated mice, enhanced communication functionality was observed in subset G3 (**Supplementary Figure 6A**). In surgery-treated mice, communication increased in subsets G0 and G3, whereas signal communication in G2 significantly decreased (**Supplementary Figure 6A**). Heatmap visualization further confirmed variations in signaling pathways across the subgroups (**Supplementary Figure 6B**). Specific ligand–receptor pairs, notably CXCL2–CXCR2, exhibited increased expression in G0, G1, and G3 after surgery and CFA injection but decreased expression in G2, G4, and G5 (**Supplementary Figures 6B–D**). Elevated levels of CXCL2 were detected in various leukocyte types post-treatment, primarily in neutrophils and basophils, while no significant change in CXCR2 expression was observed despite an increased number of CXCR2-positive cells (**Figures 4A, B**). Flow cytometry confirmed elevated CXCR2 expression between 6 and 24 hours post-surgery and between 6 and 12 hours post-CFA treatment (**Supplementary Figures 7A, B**).

Neutrophil degranulation is known to be associated with their clustering and phagocytosis, processes that play crucial roles in the inflammatory response (23, 24). Neutrophils contain four types of granules: azurophil (primary) granules, specific (secondary) granules, gelatinase granules, and secretory vesicles (25). We analyzed the expression of relevant genes using FunctionScore software (**Supplementary Table 6**). The results revealed an increase in clusters G0 and G6 across all four types of granules (**Supplementary Figure 7C**). In particular, the secretion of azurophil granules and specific granules was significantly upregulated after surgery and CFA stimulation (**Figure 4C**). Beyond degranulation, each subgroup was assessed for additional functions, including phagocytosis, chemotaxis, activation, apoptosis, and maturation (**Figures 4D–I**; **Supplementary Figure 7D–I**). Following surgery and CFA treatment, these subgroups were enriched in functions such as cellular phagocytosis, chemotaxis, activation, apoptosis, and maturation (**Figures 4E–I**). Furthermore, we evaluated the metabolic activities necessary for the survival and activation of neutrophils, specifically glucose metabolism and lipid metabolism. Under normal conditions, glucose metabolism in each subgroup remained relatively stable, except in G5 (**Supplementary Figure 7J**). Neutrophils, which contain relatively few mitochondria, primarily rely on anaerobic glycolysis to convert glucose into pyruvate (**Supplementary Figures 7K, L**). It is well-established that oxygen utilization by neutrophils is influenced by various physiological and pathological factors. In response to traumatic or inflammatory stimuli, each subgroup exhibited distinct abnormalities in glucose metabolism (**Figures 4J–L**). Carbohydrate metabolism and lipid metabolism are closely interconnected in cellular processes. In this study, we also observed abnormal lipid metabolism in neutrophils (**Figure 4M**; **Supplementary Figure 7M**). Arachidonic acid metabolism was significantly downregulated, while triglyceride metabolism was markedly activated in neutrophils following surgical and CFA stimulation (**Figures 4N, O**). This contrasted sharply with the metabolic balance observed in the subgroups under normal conditions (**Supplementary Figures 7N, O**).

### 3.5 NAMO attenuates mechanical pain sensitization by inhibiting neutrophil differentiation and maturation

**Figure 5:**
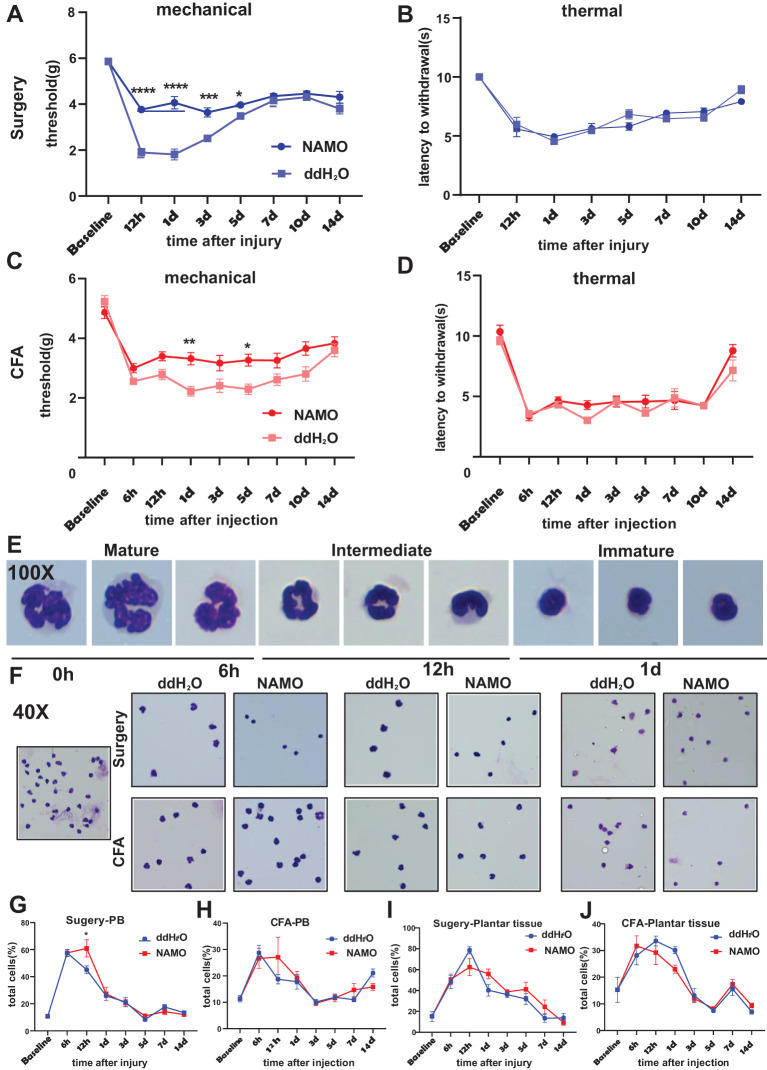
NAMO attenuates mechanical pain sensitization and delays neutrophil maturation. **(A-D)** The effects of NAMO on mechanical and thermal sensitization were evaluated following plantar incisions or CFA injections in NAMO-treated and control mice. The data are presented as the means ± SEMs (n = 8–15) and were analyzed via two-way repeated-measures ANOVA with Tukey’s *post hoc* test (*P < 0.05, **P < 0.01, ***P < 0.001, ****P < 0.0001 compared with the control). **(E, F)** Giemsa staining was performed on Ly6G^+^ cells from peripheral blood. **(E)** Neutrophils transitioned from the mature state to the naïve state (magnification 100x). **(F)** Differential smears of neutrophils from NAMO-treated and control mice revealed alterations among mature, naïve, and intermediate neutrophil forms (magnification 40x). **(G-J)** Flow cytometry analysis was used to assess the impact of NAMO on neutrophils in blood and plantar tissues across two mouse models. The data are shown as the means ± SEMs and were analyzed via two-way repeated-measures ANOVA (*P < 0.05, n = 5–6 mice per group).

NAMO has been established as a potent and selective CXCR2 antagonist, demonstrating marked inhibition of GRO-α-mediated human neutrophil chemotaxis (26, 27). Our results showed that NAMO significantly reduced mechanical hypersensitivity between 12 hours and 3 days postsurgery without affecting thermal responses (**Figures 5A, B**). In CFA-treated mice, NAMO elevated mechanical pain thresholds at 1 day and 5 days posttreatment, again with no impact on thermal responses (**Figures 5C, D**). Peripheral blood Ly6G^+^ cells from surgical or CFA-treated mice were categorized as mature, intermediate, or immature on the basis of their nuclear morphology (**Figure 5E**). In naïve mice, these cells predominantly exhibited an intermediate morphology (**Figure 5F**). At 6 hours postsurgery, neutrophils primarily displayed an intermediate to mature state, but NAMO treatment shifted them toward an immature phenotype (**Figure 5F**). After 6, 12, and 24 hours of CFA treatment, the neutrophils transitioned from an intermediate state to an immature state, with NAMO showing no significant effect on this progression (**Figure 5F**). NAMO delayed the peak elevation of neutrophils in peripheral blood from 6–12 hours posttreatment, without significantly affecting the levels in plantar tissues (**Figures 5G-J**; **Supplementary Figures 8A-D**). Furthermore, NAMO selectively modulated T cells and monocytes, delaying neutrophil differentiation and maturation between 6 and 12 hours postsurgery or after CFA treatment (**Supplementary Figures 8E-G**).

### 3.6 NAMO mitigates inflammation in plantar tissue

**Figure 6:**
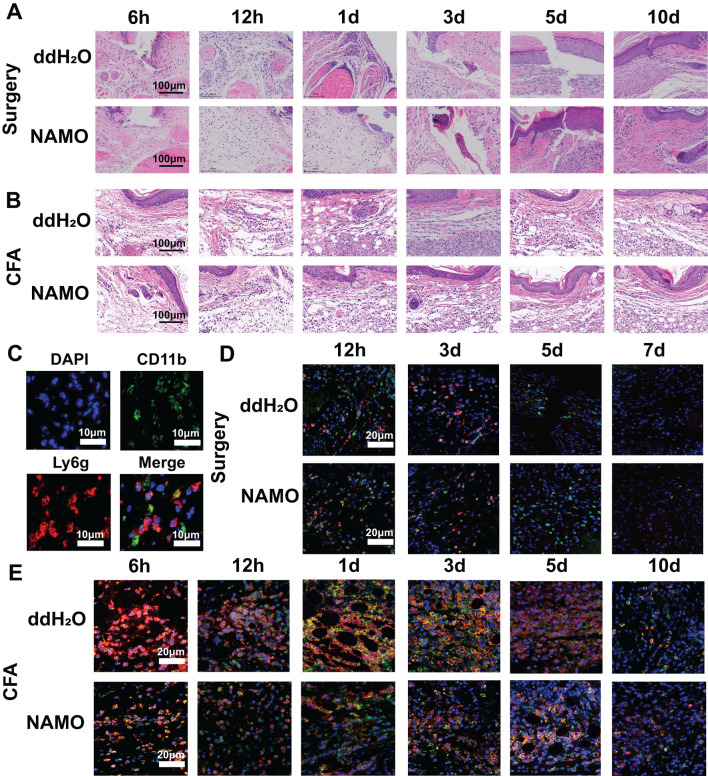
NAMO mitigates inflammation and neutrophil infiltration in plantar tissue. **(A, B)** Hematoxylin and eosin (H&E) staining was used to evaluate the inflammatory response in the plantar tissues of CFA-injected and incision-treated mice after NAMO administration. **(C-E)** Immunofluorescence staining was conducted to assess the infiltration of myeloid cells and neutrophils in the plantar tissues of CFA-injected and incision-treated mice following NAMO treatment.

Following a surgical incision in plantar tissue, inflammatory cell infiltration was observed at 6 hours postincision, peaked on day 1, decreased by day 3, and resolved by day 10 (**Figure 6A**). Conversely, CFA injection resulted in increased inflammatory infiltration at 12 hours, peaking on day 3, and persisting until day 10, which was characterized by hyperplasia, increased fat vesicle presence, and tissue laxity, especially on day 1 (**Figure 6B**). NAMO significantly inhibited neutrophil infiltration from 6 hours to 3 days under both conditions (**Figures 6C-E**).

**Figure 7:**
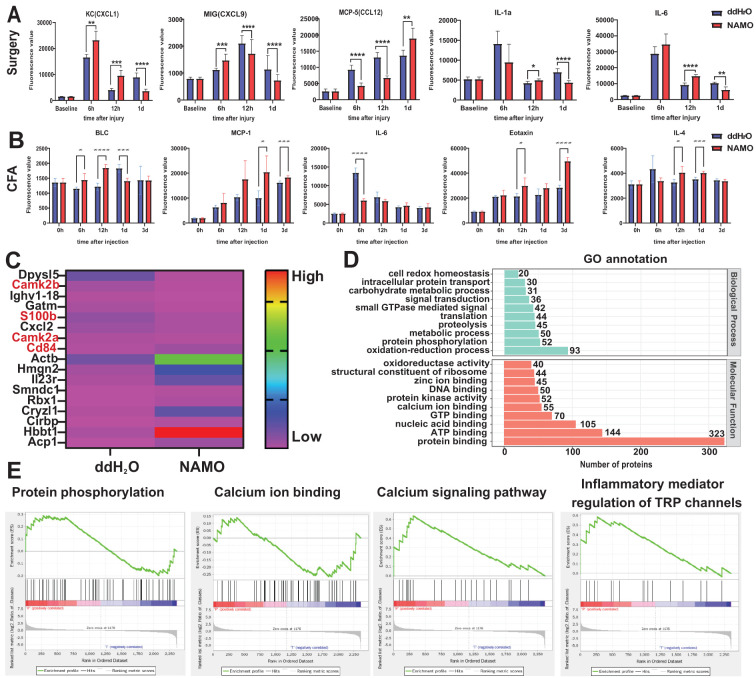
Modulation of inflammation-associated cytokine and neutrophil protein expression by the NAMO. **(A, B)** Multiplex cytokine analyses were performed on plantar tissue following CFA injection and incisional treatment, both prior to and following NAMO administration (n = 8). The data were analyzed via two-way repeated-measures ANOVA (*P < 0.05, **P < 0.01, ***P < 0.001, ****P < 0.0001 compared with the control group). **(C, E)** Label-free proteomic analysis was conducted on Ly6G^+^ cells in the peripheral blood of CFA-treated mice. **(C)** A heatmap illustrates differential protein expression between the treatment groups. **(D, E)** Gene Ontology (GO) and gene set enrichment analysis (GSEA) were performed on differentially expressed proteins (DEPs) in Ly6G^+^ cells from CFA-treated mice.

A fluorescence-based quantitative protein microarray analysis identified 40 inflammation-associated cytokines in plantar tissues following surgery or CFA injection. Surgical incisions elevated the levels of CXCL1, CXCL9, and CCL12, whereas NAMO significantly reduced the levels of these chemokines, particularly CXCL1 (**Figure 7A**). The proinflammatory cytokines IL-1α and IL-6 were upregulated at 12 hours and subsequently downregulated on day 1 with NAMO treatment (**Figure 7A**). In CFA-treated mice, the levels of BLC (CXCL13), MCP-1 (CCL2), and eotaxin (CCL11) significantly increased (**Figure 7B**). NAMO promoted MCP-1 and eotaxin expression but inhibited BLC. Although IL-6 levels rose rapidly after CFA injection, they were significantly reduced by NAMO, which also induced the expression of the anti-inflammatory cytokine IL-4 during the early stages of inflammation (**Figure 7B**).

### 3.7 NAMO regulates S100b and CaMKIIβ protein expression in Ly6G^+^ cells

Ly6G^+^ cells from CFA-treated mice, with or without NAMO intervention, were subjected to label-free proteomic analysis. Among the 247 differentially expressed proteins (DEPs), 122 were upregulated and 125 were downregulated following NAMO treatment (**Supplementary Table 7**). Notably, CD84, Hbbt1, and Cryzl1 were upregulated, whereas S100b, CaMKIIβ, and CaMKIIα were downregulated (**Figure 7C**). Gene Ontology (GO) enrichment analysis indicated that NAMO influences redox reactions, protein phosphorylation, metabolism, translation, and small GTPase-mediated signaling in Ly6G^+^ cells (**Figure 7D**). Moreover, NAMO modulates interactions between proteins and nucleic acids, ATP, protein kinases, and metal ions (**Figure 7D**). Gene set enrichment analysis (GSEA) demonstrated that NAMO substantially activated pathways related to protein phosphorylation, calcium ion signaling, and TRP channel regulation (**Figure 7E**).

**Figure 8:**
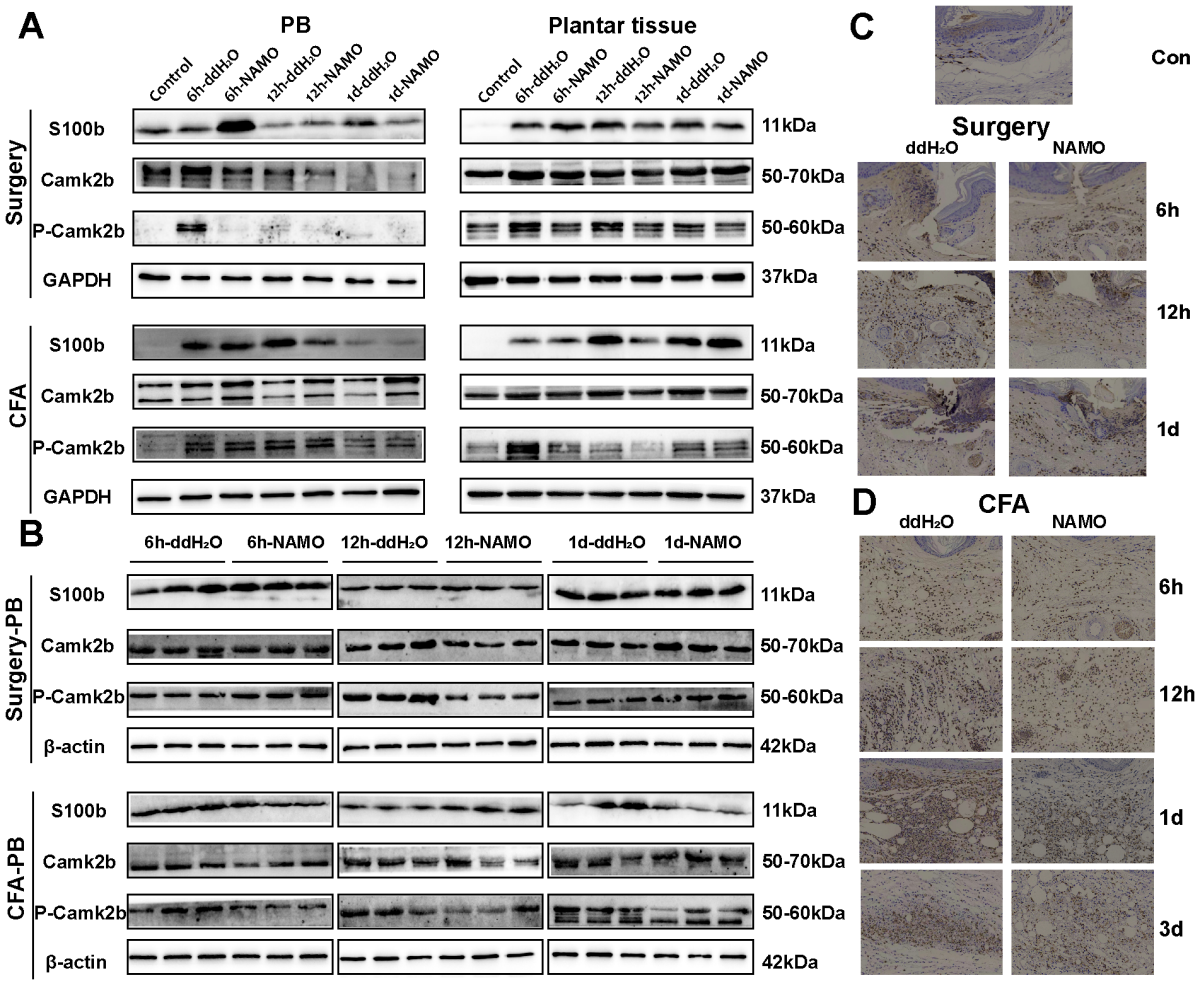
Regulation of S100b and CaMKIIβ protein expression by NAMO. **(A, B)** Western blot analysis was used to measure the protein expression levels of S100b and CaMKIIβ in peripheral blood, plantar tissue, and Ly6G^+^ cells from mice that underwent surgery and CFA treatment, both before and after NAMO administration. **(C, D)** Immunohistochemical analysis was used to assess S100b protein expression in the plantar tissues of surgically treated and CFA-treated mice both before and after NAMO treatment. Cells with positive staining are indicated by a dark brown color (scale bar = 100 μm).

S100b, CaMKIIβ, and phosphorylated CaMKIIβ (pCaMKIIβ) were minimally expressed in naïve mice but were upregulated following surgical and CFA treatment (**Figure 8A**). In Ly6G^+^ cells, the expression patterns of CaMKIIβ and S100b in incised mice corresponded with those in peripheral blood, whereas CFA-treated mice presented different S100b expression patterns (**Figure 8B**). Post-NAMO administration, S100b expression increased in surgery-treated mice at 6 hours but decreased in CFA-treated mice at 12 hours (**Figures 8A, C, D**). The levels of pCaMKIIβ decreased in both the blood and plantar tissues of surgery- and CFA-treated mice from 6–12 hours (**Figure 8A**). These observations indicate that NAMO mitigates inflammation and pain by modulating the expression of S100b, CaMKIIβ, and pCaMKIIβ in neutrophils.

## 4 Discussion

In this study, we employed single-cell RNA sequencing to analyze CD11b^+^ myeloid cells, focusing on their molecular characteristics and intercellular signaling during inflammation induced by surgery and CFA. The results revealed an increase in the number of neutrophils and a decrease in the number of monocytes among the CD11b^+^ cells in both models. The majority of the increased neutrophils were mature, characterized by high expression of CD177 and CXCR2, and depended on the CXCL signaling pathway. Despite the reduction in monocyte numbers, their roles in leukocyte chemotaxis, migration, differentiation, and signaling remain pronounced, particularly through pathways dependent on CD92 and CD52 in naïve monocytes (M3). CXCR2, a marker for mature neutrophils, is involved in sensory pain sensitivity due to early tissue injury and inflammation. Our findings indicate that NAMO significantly reduces mechanical nociception by delaying neutrophil differentiation and maturation in surgically treated and CFA-treated mice without affecting thermal nociception. Furthermore, NAMO modulates the protein levels of CaMK and S100b in neutrophils following surgery and CFA injection, indicating that these proteins are involved in early-stage postoperative and CFA-induced inflammatory pain models.

A surgical incision acts as a traumatic stimulus, triggering both systemic neuroendocrine and local inflammatory responses that activate the sympathetic nervous system. This trauma initiates an acute phase response to mitigate tissue damage, combat infection, and begin healing processes (28–30). However, inflammation and pain are intricately connected, as proinflammatory cytokines at nerve injury sites contribute to central sensitization and neuropathic pain (31). Neutrophils, the most prevalent type of white blood cells in humans, play a pivotal role in acute inflammation, and their relative abundance serves as a promising indicator for detecting post-operative infections (32–34). An elevated neutrogranulo-lymphocyte ratio (NLR) is significantly correlated with the occurrence of chronic postsurgical pain (CPSP) following abdominal surgery, thoracotomy, and spinal procedures (6, 35, 36). This study revealed a significant increase in the number of peripheral blood neutrophils and a decrease in the number of lymphocytes in surgical and CFA-induced pain models. The observed trends in myeloid-derived inflammatory cells mirrored the fluctuations in pain, suggesting a strong association between these cells and the dynamics of pain.

Diverse outcomes from studies on neutrophil depletion suggest a regulatory function in pain sensitization. Specifically, the depletion of circulating neutrophils reduces early pain sensitization following peripheral nerve injury. Inhibiting neutrophil infiltration via the migration inhibitor fucoidan alleviates mechanical hyperalgesia following carrageenan injection in rat hind paws (37). In a CFA-induced inflammatory pain model, neutrophil-derived opioids decrease pain sensitivity, although neutrophil depletion does not affect baseline sensitization, potentially facilitating endogenous peripheral analgesia. Nonetheless, inconsistent results from neutrophil depletion studies complicate the elucidation of their specific role in postoperative pain (38–40). In the present study, single-cell sequencing of neutrophils from postoperative and CFA-induced inflammatory pain models revealed a significant increase in the neutrophil population, which was categorized into seven subpopulations on the basis of transcriptomic characteristics, reflecting different stages of differentiation. Early-stage neutrophils demonstrate strong antibacterial activity, with proinflammatory effects intensifying as they mature. These findings suggest that the role of neutrophils at various stages of differentiation in postoperative pain merits further investigation. Neutrophils in both postoperative and CFA-induced inflammatory pain models coexpressed Ly6G and CD14 at 12 hours postinjury, with significant infiltration of Ly6G^+^CD14^+^ cells observed in mouse paw tissue. Prior studies have indicated that Ly6G^+^CD14^+^ cell subsets promote nerve repair and axon regeneration (41), suggesting their potential involvement in the inflammatory process and pain sensitization. Peripheral blood analyses revealed that changes in Ly6G^+^CD14^+^ cell populations correlate with alterations in mechanical and thermal pain thresholds in mice. Given their role in nerve repair and axon regeneration, further exploration into the involvement of Ly6G^+^CD14^+^ cells in wound healing and inflammatory pain is warranted.

NAMO influences the expression of various chemokines (e.g., CXCL1, CXCL9, CXCL13, CCL12, and CCL17), interleukins (e.g., IL-1α, IL-4, and IL-6), and inflammatory factors (e.g., TNF-α, leptin, and TIMP-1) within mouse paw tissues during the postoperative and early inflammatory stages, thereby mitigating tissue inflammation. While they are traditionally associated with immune cell secretion, our study links the expression of cytokines and chemokines to ligand–receptor signaling pathways on immune cell surfaces. CXCL1 (KC), which is secreted by macrophages, neutrophils, and epithelial cells, serves as a principal ligand for CXCR2. CXCL9 (MIG), an ELR-negative CXC chemokine induced by IFN-γ, recruits CXCR3^+^ cells (e.g., effector T cells, regulatory T cells, and CD8^+^ cytotoxic T cells) via the CXCR3 receptor (42). Although the expression of CXCL9 in spinal astrocytes significantly increases following spinal nerve ligation (SNL) (43, 44), the intrathecal injection of CXCL9 does not induce hyperalgesia or pain, possibly because of its dual role in enhancing excitatory and inhibitory synaptic transmission. CCL12 (MCP-5) functions as a potent chemoattractant for peripheral blood monocytes, attracting eosinophils, monocytes, and lymphocytes but not neutrophils, and induces calcium flux in peripheral blood mononuclear cells. CCL12 signals through the CCR2 receptor, playing a critical role in allergic inflammation and the pathogen response, and is important in the early stages of allergic lung inflammation (45).

Our study revealed a significant increase in the expression of both CXCL1 and CXCR2 following surgical intervention, indicating that activation of the CXCL1-CXCR2 signaling pathway is a response to tissue injury. Treatment with NAMO, which reduces the number of CXCR2 receptor binding sites, was associated with a notable increase in free CXCL1 levels. Similarly, although CXCL9 and CCL12 levels were elevated postsurgery, they decreased significantly following NAMO treatment. These observations suggest that CXCL9 and CCL12 are integral to postoperative inflammation and pain and that NAMO exerts inhibitory effects on neutrophil differentiation and maturation, as well as on the recruitment of T cells and monocytes into plantar tissue. Such inhibition potentially mitigates the infiltration of inflammatory cells postoperatively. These findings not only increase our understanding of these inflammatory mediators but also identify novel targets and directions for future research on inflammation and pain following tissue injury.

S100b, a protein specific to the central nervous system and part of the S100 family, is predominantly produced by astrocytes. The S100 protein family, comprising 25 members, regulates diverse cellular functions upon Ca²^+^ binding, including proliferation, differentiation, inflammation, migration, invasion, apoptosis, calcium homeostasis, and energy metabolism (46). S100b is associated with cancers, as well as inflammatory and immune diseases; its levels fluctuate following brain injury or disruption of the blood–brain barrier (47). Electroacupuncture has been shown to attenuate CFA-induced inflammatory pain by suppressing Nav1.8 through S100b in mice (48). CaMKII modulates both physiological and behavioral activities. It forms a tetramer consisting of α, β, γ, and δ subunits, each containing catalytic and regulatory domains (49). CaMKIIα, which is located primarily in the hippocampus and cerebral cortex, is crucial for learning and memory (50). Moreover, CaMKIIα can facilitate the transition from acute to chronic pain by inducing nociceptive hypersensitivity (51). In addition to being expressed in the brain, CaMKIIβ is expressed in skeletal muscle and the pancreas (52). Recent studies have demonstrated that the CaMKIIβ pathway promotes astrocyte proliferation in spinal cord injury by regulating the inflammatory factor IL-1α (53).CaMKIIγ has been closely linked to colon cancer (54), whereas CaMKIIδ plays a significant role in osteoarthritis (55). Atypical Wnt pathways involving CAMK2 may contribute to CFA-induced chronic inflammatory nociceptive hypersensitivity (56). The mobilization of bone marrow cells, particularly neutrophils and monocytes/macrophages, into the peripheral blood following surgery or infection, along with their recruitment to injured tissues, facilitates pathogen clearance and tissue repair. Despite the increase in neutrophils being a focal point of pain research, the depletion of neutrophils with anti-Ly6G monoclonal antibodies has proven ineffective for pain relief. This study proposes the use of a CXCR2 antagonist to inhibit neutrophil differentiation and maturation, warranting further mechanistic and clinical investigations for the prevention and treatment of inflammatory pain.

## Data Availability

The data presented in the study are deposited in the SRA database, accession number: PRJNA1265861.

## References

[1] Mackey S. Mechanisms of inflammatory pain. JCR: J Clin Rheumatol. (2004) 10:S5–S11. doi: 10.1097/01.rhu.0000130684.35729.55 17043503

[2] Giraud F Pereira E Anizon F Moreau P. Recent advances in pain management: relevant protein kinases and their inhibitors. Molecules. (2021) 26:2696. doi: 10.3390/molecules26092696 34064521 PMC8124620

[3] Carvalho NS Lemes JBP Pagliusi M Jr MaChado A Malange KF Pral LP. Neutrophil-Derived COX-2 has a Key Role during Inflammatory Hyperalgesia. Inflammation. (2022) 45:2280–93. doi: 10.1007/s10753-022-01690-5 35840810

[4] Baral P Udit S Chiu IM. Pain and immunity: implications for host defence. Nat Rev Immunol. (2019) 19:433–47. doi: 10.1038/s41577-019-0147-2 PMC6700742 30874629

[5] Hiroki CH Yipp BG. Neutrophils are itching to specialize. Immunity. (2024) 57:198–200. doi: 10.1016/j.immuni.2024.01.012 38354698

[6] Shu B Xu F Zheng X Zhang Y Liu Q Li S. Change in perioperative neutrophil-lymphocyte ratio as a potential predictive biomarker for chronic postsurgical pain and quality of life: an ambispective observational cohort study. Front Immunol. (2023) 14:1177285. doi: 10.3389/fimmu.2023.1177285 37122722 PMC10130394

[7] Ghasemlou N Chiu IM Julien J-P Woolf CJ. CD11b+Ly6G–myeloid cells mediate mechanical inflammatory pain hypersensitivity. Proc Natl Acad Sci. (2015) 112:E6808–17. doi: 10.1073/pnas.1501372112 PMC4679057 26598697

[8] Cunha TM Barsante MM Guerrero AT Verri WA Ferreira SH Coelho FM. Treatment with DF 2162, a non-competitive allosteric inhibitor of CXCR1/2, diminishes neutrophil influx and inflammatory hypernociception in mice. Br J Pharmacol. (2009) 154:460–70. doi: 10.1038/bjp.2008.94 PMC2442455 18362895

[9] Carreira EU Carregaro V Teixeira MM Moriconi A Aramini A Verri WA. Neutrophils recruited by CXCR1/2 signalling mediate post-incisional pain. Eur J Pain. (2012) 17:654–63. doi: 10.1002/j.1532-2149.2012.00240.x 23132735

[10] Sahbaie P Li X Shi X Clark JD. Roles of gr-1+Leukocytes in postincisional nociceptive sensitization and inflammation. Anesthesiology. (2012) 117:602–12. doi: 10.1097/ALN.0b013e3182655f9f PMC3427475 22820848

[11] Petrović J Silva JR Bannerman CA Segal JP Marshall AS Haird CM. γδ T cells modulate myeloid cell recruitment but not pain during peripheral inflammation. Front Immunol. (2019) 10:473. doi: 10.3389/fimmu.2019.00473 30936874 PMC6431614

[12] Basso L Boué J Bourreille A Dietrich G. Endogenous regulation of inflammatory pain by T-cell-derived opioids. Inflammatory Bowel Diseases. (2014) 20:1870–7. doi: 10.1097/mib.0000000000000073 24846722

[13] Mitsui K Hishiyama S Jain A Kotoda Y Abe M Matsukawa T. Role of macrophage autophagy in postoperative pain and inflammation in mice. J Neuroinflammation. (2023) 20:102. doi: 10.1186/s12974-023-02795-w 37131209 PMC10152627

[14] Oliveira SM Drewes CC Silva CR Trevisan G Boschen SL Moreira CG. Involvement of mast cells in a mouse model of postoperative pain. Eur J Pharmacol. (2011) 672:88–95. doi: 10.1016/j.ejphar.2011.10.001 22004612

[15] Raymondi Silva J Iftinca M Fernandes Gomes FI Segal JP Smith OMA Bannerman CA. Skin-resident dendritic cells mediate postoperative pain via CCR4 on sensory neurons. Proc Natl Acad Sci. (2022) 119:e2118238119. doi: 10.1073/pnas.2118238119 35046040 PMC8794894

[16] Dai H Li L Zeng T Chen L. Cell-specific network constructed by single-cell RNA sequencing data. Nucleic Acids Res. (2019) 47:e62–2. doi: 10.1093/nar/gkz172 PMC6582408 30864667

[17] Sadler KE Mogil JS Stucky CL. Innovations and advances in modelling and measuring pain in animals. Nat Rev Neurosci. (2021) 23:70–85. doi: 10.1038/s41583-021-00536-7 34837072 PMC9098196

[18] Sukeishi A Isami K Hiyama H Imai S Nagayasu K Shirakawa H. Colchicine alleviates acute postoperative pain but delays wound repair in mice: Roles of neutrophils and macrophages. Mol Pain. (2017) 13:1744806917743680. doi: 10.1177/1744806917743680 29108466 PMC5692123

[19] Wedel S Osthues T Zimmer B Angioni C Geisslinger G Sisignano M. Oxidized linoleic acid metabolites maintain mechanical and thermal hypersensitivity during sub-chronic inflammatory pain. Biochem Pharmacol. (2022) 198:114953. doi: 10.1016/j.bcp.2022.114953 35149052

[20] Blin-Wakkach C Wakkach A Quincey D Carle GF. Interleukin-7 partially rescues B-lymphopoiesis in osteopetrotic oc/oc mice through the engagement of B220+CD11b+ progenitors. Exp Hematol. (2006) 34:851–9. doi: 10.1016/j.exphem.2006.04.003 16797412

[21] Wagner C Hänsch GM Stegmaier S Denefleh B Hug F Schoels M. The complement receptor 3, CR3 (CD11b/CD18), on T lymphocytes: activation-dependent up-regulation and regulatory function. Eur J Immunol. (2001) 31:1173–80. doi: 10.1002/1521-4141(200104)31:4<1173::Aid-immu1173>3.0.Co;2-9 11298342

[22] Ligeron C Saenz J Evrard B Drouin M Merieau E Mary C. CLEC-1 restrains acute inflammatory response and recruitment of neutrophils following tissue injury. J Immunol. (2024) 212:1178–87. doi: 10.4049/jimmunol.2300479 38353642

[23] Xie X Shi Q Wu P Zhang X Kambara H Su J. Single-cell transcriptome profiling reveals neutrophil heterogeneity in homeostasis and infection. Nat Immunol. (2020) 21:1119–33. doi: 10.1038/s41590-020-0736-z PMC7442692 32719519

[24] Huang J Zhu Z Ji D Sun R Yang Y Liu L. Single-cell transcriptome profiling reveals neutrophil heterogeneity and functional multiplicity in the early stage of severe burn patients. Front Immunol. (2022) 12:792122. doi: 10.3389/fimmu.2021.792122 35116026 PMC8803731

[25] Borregaard N Sørensen OE Theilgaard-Mönch K. Neutrophil granules: a library of innate immunity proteins. Trends Immunol. (2007) 28:340–5. doi: 10.1016/j.it.2007.06.002 17627888

[26] Khlebnikov AI Schepetkin IA Quinn MT. Quantitative structure–activity relationships for small non-peptide antagonists of CXCR2: Indirect 3D approach using the frontal polygon method. Bioorganic Medicinal Chem. (2006) 14:352–65. doi: 10.1016/j.bmc.2005.08.026 16182534

[27] Cutshall NS Ursino R Kucera KA Latham J Ihle NC. Nicotinamide N-oxides as CXCR2 antagonists. Bioorg Med Chem Lett. (2001) 11:1951–4. doi: 10.1016/s0960-894x(01)00326-2 11459668

[28] Pak DJ Yong RJ Kaye AD Urman RD. Chronification of pain: mechanisms, current understanding, and clinical implications. Curr Pain Headache Reports. (2018) 22:9. doi: 10.1007/s11916-018-0666-8 29404791

[29] Pogatzki-Zahn EM Segelcke D Schug SA. Postoperative pain—from mechanisms to treatment. Pain Reports. (2017) 2:e588. doi: 10.1097/pr9.0000000000000588 29392204 PMC5770176

[30] Gulur P Nelli A. Persistent postoperative pain. Curr Opin Anaesthesiol. (2019) 32:668–73. doi: 10.1097/aco.0000000000000770 31343465

[31] Powell R Young VA Pryce KD Sheehan GD Bonsu K Ahmed A. Inhibiting endocytosis in CGRP+ nociceptors attenuates inflammatory pain-like behavior. Nat Communications. (2021) 12:5812. doi: 10.1038/s41467-021-26100-6 PMC8490418 34608164

[32] Chu D Dong X Shi X Zhang C Wang Z. Neutrophil-based drug delivery systems. Advanced Materials. (2018) 30:e1706245. doi: 10.1002/adma.201706245 29577477 PMC6161715

[33] Qian B Zheng Y Jia H Zheng X Gao R Li W. Neutrophil-lymphocyte ratio as a predictive marker for postoperative infectious complications: A systematic review and meta-analysis. Heliyon. (2023) 9:e15586. doi: 10.1016/j.heliyon.2023.e15586 37159687 PMC10163603

[34] Amulic B Cazalet C Hayes GL Metzler KD Zychlinsky A. Neutrophil function: from mechanisms to disease. Annu Rev Immunol. (2012) 30:459–89. doi: 10.1146/annurev-immunol-020711-074942 22224774

[35] Semerci T Tugcugil E Besir A. Relación entre la proporción neutrófilos/linfocitos con el momento de la analgesia epidural y el dolor de la toracotomía. Cirugía y Cirujanos. (2024) 92:33–8. doi: 10.24875/ciru.23000161 38537235

[36] Inose H Kobayashi Y Yuasa M Hirai T Yoshii T Okawa A. Procalcitonin and neutrophil lymphocyte ratio after spinal instrumentation surgery. Spine. (2019) 44:E1356–61. doi: 10.1097/brs.0000000000003157 31725684

[37] Cunha TM Verri WA Schivo IR Napimoga MH Parada CA Poole S. Crucial role of neutrophils in the development of mechanical inflammatory hypernociception. J Leukocyte Biol. (2008) 83:824–32. doi: 10.1189/jlb.0907654 18203872

[38] Brack A Rittner HL Machelska H Leder K Mousa SA Schäfer M. Control of inflammatory pain by chemokine-mediated recruitment of opioid-containing polymorphonuclear cells. Pain. (2004) 112:229–38. doi: 10.1016/j.pain.2004.08.029 15561377

[39] Rittner HL Hackel D Yamdeu RS Mousa SA Stein C Schäfer M. Antinociception by neutrophil-derived opioid peptides in noninflamed tissue—Role of hypertonicity and the perineurium. Brain Behavior Immunity. (2009) 23:548–57. doi: 10.1016/j.bbi.2009.02.007 19233260

[40] Rittner HL Mousa SA Labuz D Beschmann K Schäfer M Stein C. Selective local PMN recruitment by CXCL1 or CXCL2/3 injection does not cause inflammatory pain. J Leukocyte Biol. (2006) 79:1022–32. doi: 10.1189/jlb.0805452 16522746

[41] Sas AR Carbajal KS Jerome AD Menon R Yoon C Kalinski AL. A new neutrophil subset promotes CNS neuron survival and axon regeneration. Nat Immunol. (2020) 21:1496–505. doi: 10.1038/s41590-020-00813-0 PMC7677206 33106668

[42] Tokunaga R Zhang W Naseem M Puccini A Berger MD Soni S. CXCL9, CXCL10, CXCL11/CXCR3 axis for immune activation – A target for novel cancer therapy. Cancer Treat Rev. (2018) 63:40–7. doi: 10.1016/j.ctrv.2017.11.007 PMC5801162 29207310

[43] Jiang B-C He L-N Wu X-B Shi H Zhang W-W Zhang Z-J. Promoted interaction of C/EBPα with demethylatedCxcr3Gene promoter contributes to neuropathic pain in mice. J Neurosci. (2017) 37:685–700. doi: 10.1523/jneurosci.2262-16.2016 28100749 PMC6596757

[44] Kong Y-F Sha W-L Wu X-B Zhao L-X Ma L-J Gao Y-J. CXCL10/CXCR3 signaling in the DRG exacerbates neuropathic pain in mice. Neurosci Bulletin. (2020) 37:339–52. doi: 10.1007/s12264-020-00608-1 PMC7955022 33196963

[45] Ma C Gao J Liang J Wang F Xu L Bu J. CCL12 induces trabecular bone loss by stimulating RANKL production in BMSCs during acute lung injury. Exp Mol Med. (2023) 55:818–30. doi: 10.1038/s12276-023-00970-w PMC10167364 37009797

[46] Michetti F D’Ambrosi N Toesca A Puglisi MA Serrano A Marchese E. The S100B story: from biomarker to active factor in neural injury. J Neurochem. (2018) 148:168–87. doi: 10.1111/jnc.14574 30144068

[47] Koh SXT Lee JKW. S100B as a marker for brain damage and blood–brain barrier disruption following exercise. Sports Med. (2013) 44:369–85. doi: 10.1007/s40279-013-0119-9 24194479

[48] Liao H-Y Hsieh C-L Huang C-P Lin Y-W. Electroacupuncture Attenuates CFA-induced Inflammatory Pain by suppressing Nav1.8 through S100B, TRPV1, Opioid, and Adenosine Pathways in Mice. Sci Rep. (2017) 7:42531. doi: 10.1038/srep42531 28211895 PMC5304170

[49] He Q Li Z. The dysregulated expression and functional effect of CaMK2 in cancer. Cancer Cell Int. (2021) 21:326. doi: 10.1186/s12935-021-02030-7 34193145 PMC8243487

[50] Chang J-Y Nakahata Y Hayano Y Yasuda R. Mechanisms of Ca2+/calmodulin-dependent kinase II activation in single dendritic spines. Nat Communications. (2019) 10:2784. doi: 10.1038/s41467-019-10694-z PMC6592955 31239443

[51] Ferrari LF Bogen O Levine JD. Role of nociceptor caMKII in transition from acute to chronic pain (Hyperalgesic priming) in male and female rats. J Neurosci. (2013) 33:11002–11. doi: 10.1523/jneurosci.1785-13.2013 PMC3718370 23825405

[52] Cook SG Bourke AM O’Leary H Zaegel V Lasda E Mize-Berge J. Analysis of the CaMKIIα and β splice-variant distribution among brain regions reveals isoform-specific differences in holoenzyme formation. Sci Reports. (2018) 8:5448. doi: 10.1038/s41598-018-23779-4 PMC5882894 29615706

[53] Xia Y Ding L Zhang C Xu Q Shi M Gao T. Inflammatory factor IL1α Induces aberrant astrocyte proliferation in spinal cord injury through the grin2c/ca2+/caMK2b pathway. Neurosci Bulletin. (2023) 40:421–38. doi: 10.1007/s12264-023-01128-4 PMC11003951 37864744

[54] Ma X Meng Z Jin L Xiao Z Wang X Tsark WM. CAMK2γ in intestinal epithelial cells modulates colitis-associated colorectal carcinogenesis via enhancing STAT3 activation. Oncogene. (2017) 36:4060–71. doi: 10.1038/onc.2017.16 PMC5509478 28319059

[55] Zhang X Wang C Zhao J Xu J Geng Y Dai L. miR-146a facilitates osteoarthritis by regulating cartilage homeostasis via targeting Camk2d and Ppp3r2. Cell Death Disease. (2017) 8:e2734–4. doi: 10.1038/cddis.2017.146 PMC5477577 28383548

[56] Lu Y Liu M Guo X Wang P Zeng F Wang H. miR-26a-5p alleviates CFA-induced chronic inflammatory hyperalgesia through Wnt5a/CaMKII/NFAT signaling in mice. CNS Neurosci Ther. (2023) 29:1254–71. doi: 10.1111/cns.14099 PMC10068476 36756710

