# Supplementary material for: Exploring the role of neutrophils in inflammatory pain hypersensitivity via single-cell transcriptome profiling
Source: Front Immunol. 2025 May 28;16:1552993. doi: 10.3389/fimmu.2025.1552993 (PMC12151834; doi:10.3389/fimmu.2025.1552993)
Supplement: Supplementary file 1 [file DataSheet1.pdf]

Supplementary Material

1 Supplementary Figures

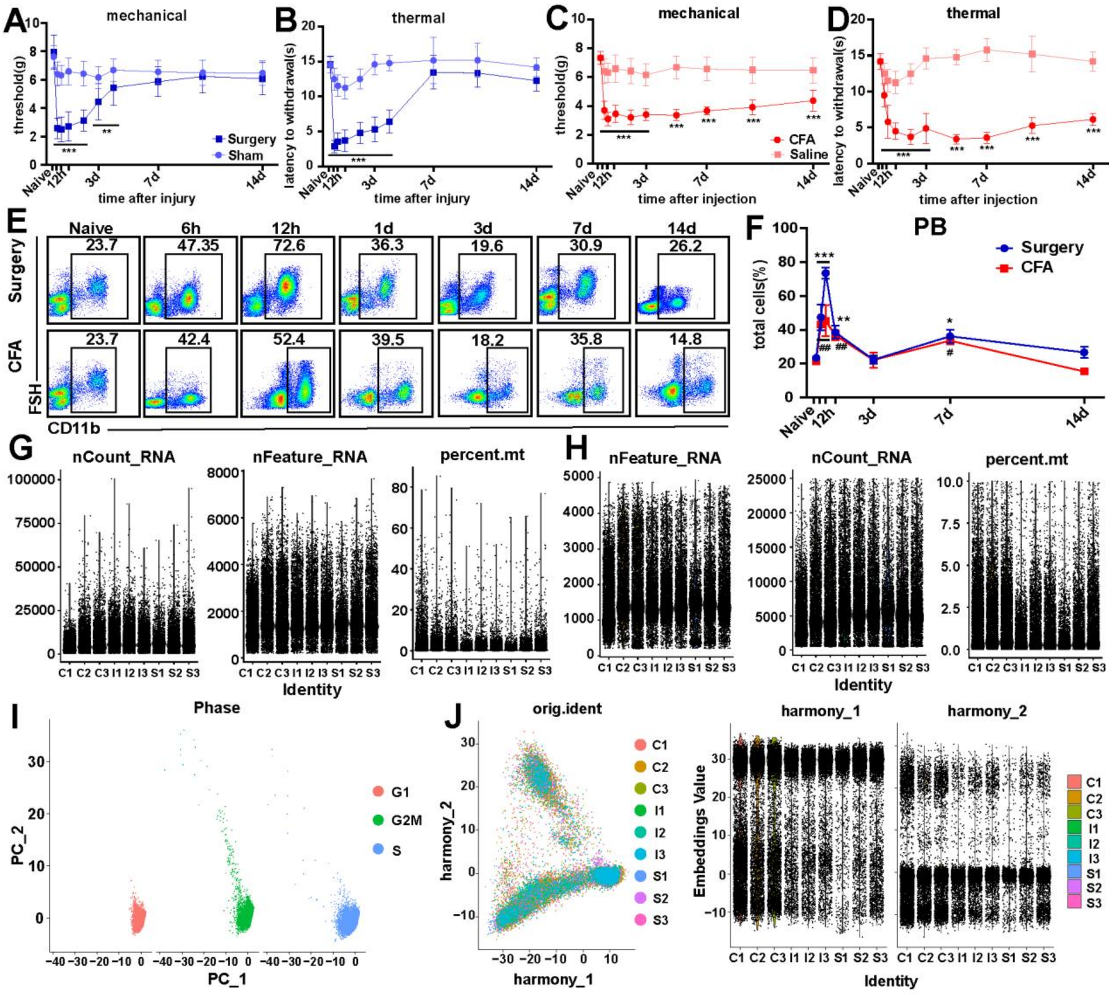

Supplementary Figure1. Analysis of mechanical and thermal hypersensitivity and immune responses in naïve, surgical, and CFA-treated mice. (A-B) Mechanical and thermal hypersensitivity were induced in C57BL/6J mice following a plantar incision. (C-D) Post-CFA injection into the plantar fascia, hypersensitivity was assessed via von Frey monofilaments (n=15, two-way ANOVA with Tukey's post hoc test; **P < 0.01, ***P < 0.001). (E-F) Flow cytometry was used to detect fluctuations in CD11b+ cells in the peripheral blood after surgical incision or CFA injection. The data are the means ± SDs. Incision: ***P < 0.001, **P < 0.01; CFA: ##P < 0.01, #P < 0.05; one-way RM-ANOVA; n=5 for incisions, n=3 for CFAs. (G-H) Violin plots of the

number of genes, number of UMIs, and percentage of mitochondrial content in nine samples before and after quality control. (I) Cell cycle phase distribution across all samples. No significant inconsistencies were detected, ensuring reliability in subsequent analyses. (J) The Harmony algorithm iteratively removes batch effects and technical noise from the nine samples, increasing sequencing data quality. Only the first two dimensions are illustrated.

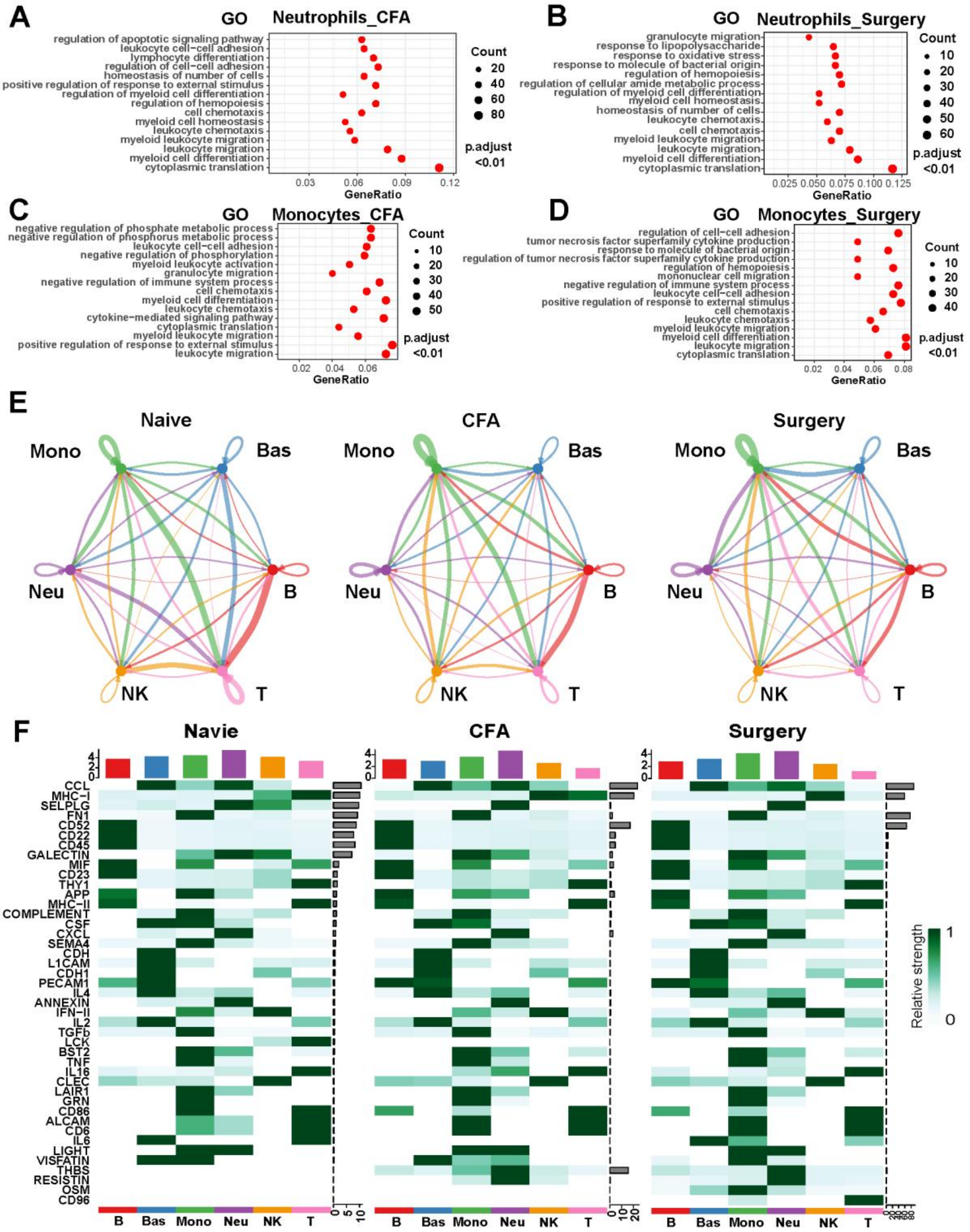

Supplementary Figure2. Analysis of CD11b+ cell function in three mouse models. (A-D) Gene Ontology (GO) analysis of differentially expressed genes (DEGs) in neutrophil and monocyte subsets from surgery- or CFA-treated mice. Selected GO terms with corrected P values < 0.05 are shown. (E) Network visualization of cell–cell communication among immune cells in the three models via the KEGG dataset. The edge width indicates the communication strength, and the arrow direction represents the signal flow. (F) Heatmaps displaying signaling pathways involved in immune cell communication in the three models. The top color bars indicate the overall signaling pathway intensity, whereas the gray bars on the right reflect the pathway expression intensity across immune cells. Abbreviations: Neu: neutrophils; Mono: monocytes; B: B lymphocytes; T: T lymphocytes; Bas: basophils; NK: natural killer cells.

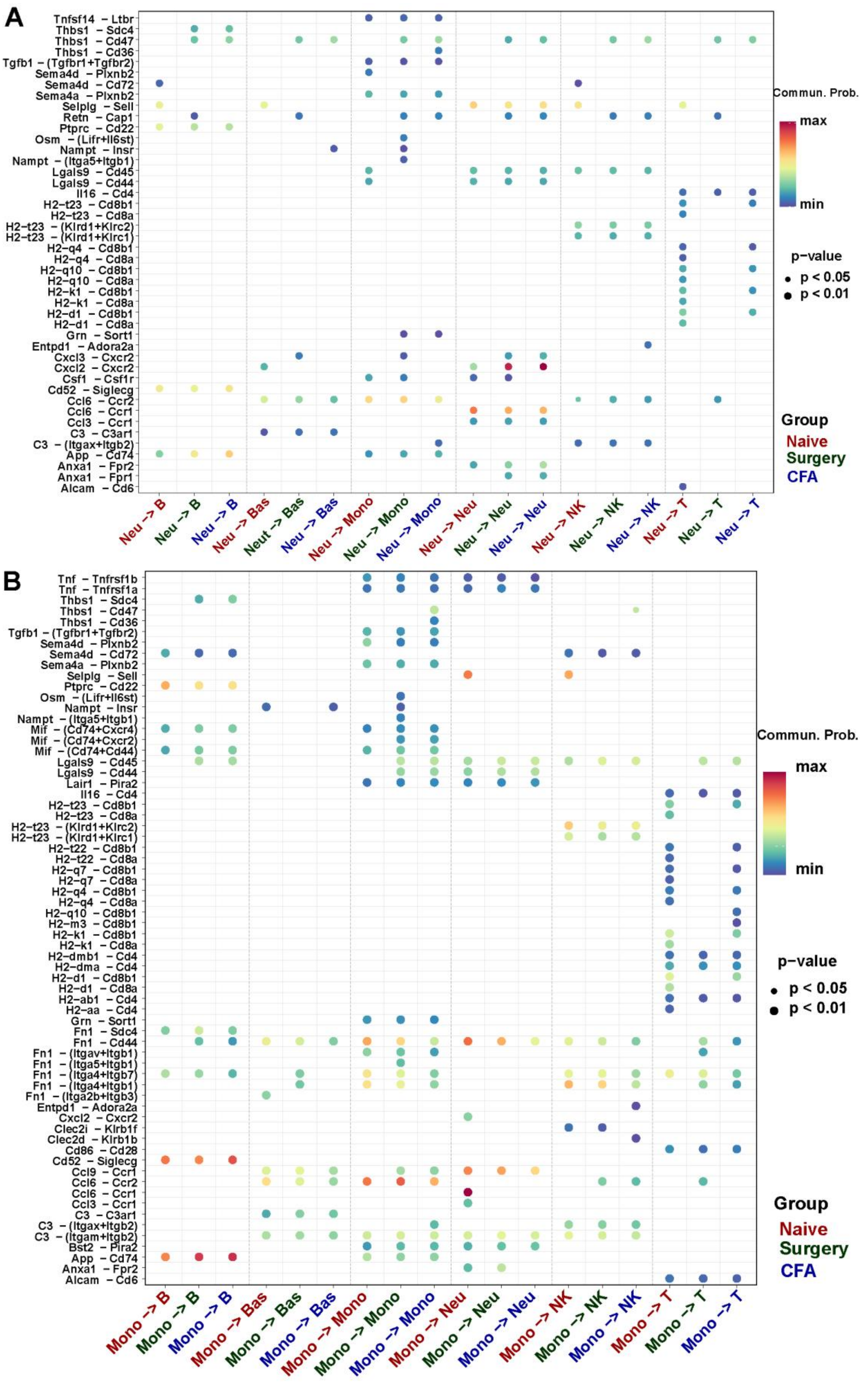

Supplementary Figure3. Differences in ligand–receptor interaction strengths. (A-B) In all three models, neutrophils and monocytes act as signal transmitters to other cell subpopulations. The dot size represents the P value (larger dots for smaller P values),

and the dot color indicates the ligand–receptor interaction strength (blue to red indicates downregulation to upregulation). (A) The CXCL2-CXCR2 pathway, notable in neutrophil self-interaction, is significantly upregulated in surgical and CFA-treated mice compared with naïve mice. (B) Compared with those in naïve mice, monocyte–neutrophil interactions via CCL6–CCR1 are significantly downregulated, whereas App–CD74 interactions are significantly upregulated in monocytes and neutrophils and basophils in surgical- and CFA-treated mice.

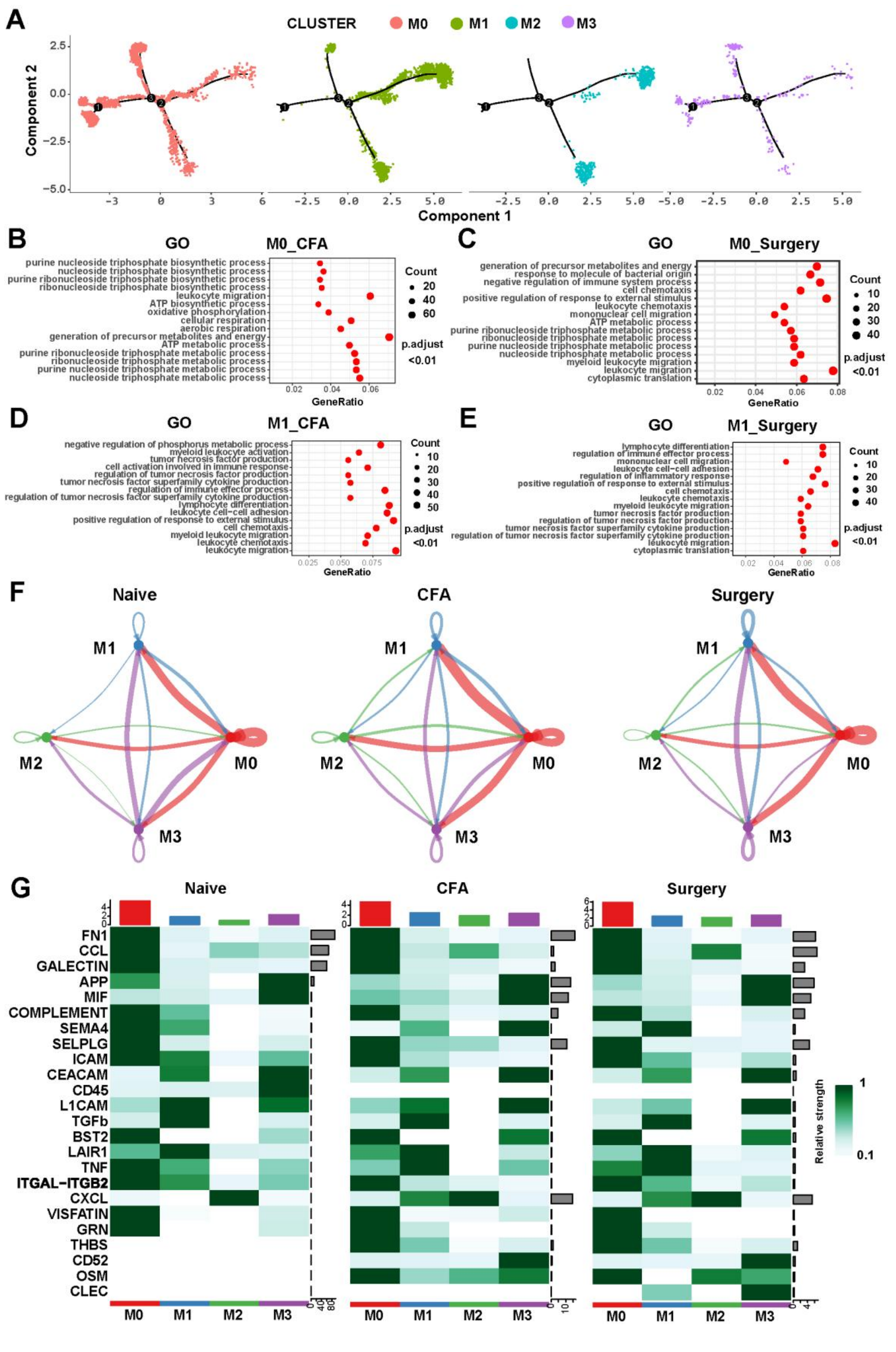

Supplementary Figure4. Monocyte function in mouse pain models. (A) Monocle trajectories of monocytes differentiated by subsets. (B-E) GO analysis of differentially expressed genes (DEGs) in the M0 and M1 clusters from surgery- or CFA-treated mice, with selected GO terms (corrected P < 0.05) displayed. (F)

Network visualization of monocyte subset communication in the three models via the KEGG dataset. The edge width denotes the communication strength, with arrows indicating the signal direction. (G) Heatmaps of signaling pathways associated with monocyte subset communication in the three models. The top color bar indicates the overall pathway expression intensity for monocyte subsets, whereas the gray bar shows the pathway expression intensity across all subsets.

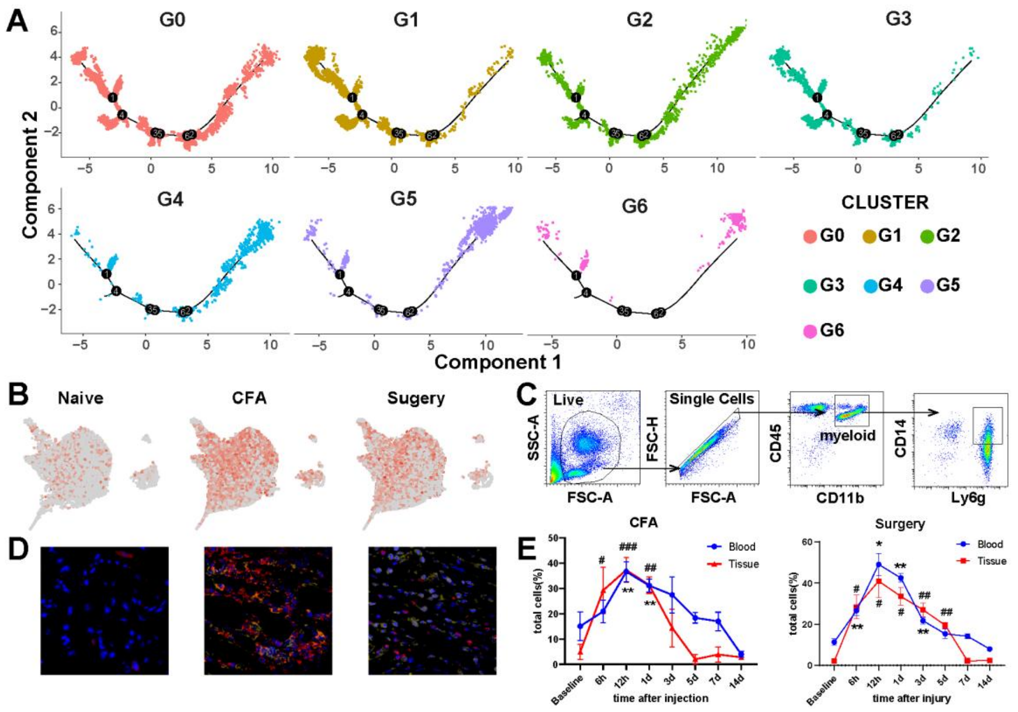

Supplementary Figure 5. Neutrophil pseudotemporal trajectory and subpopulation analysis. (A) Monocle trajectories of neutrophil subsets. (B, D) UMAP and immunofluorescence of CD14 expression in naïve, surgical, and CFA-treated mouse neutrophils. Blue: DAPI; pink: Ly6G; red: CD14. (C) FCM protocol for Ly6G^{+}CD14^{+} cell detection. (E) FCM-verified Ly6G^{+}CD14^{+} expression in peripheral blood and posterior plantar tissue. The data are presented as the means ± SDs (n=3–6 mice per group) from two independent experiments.

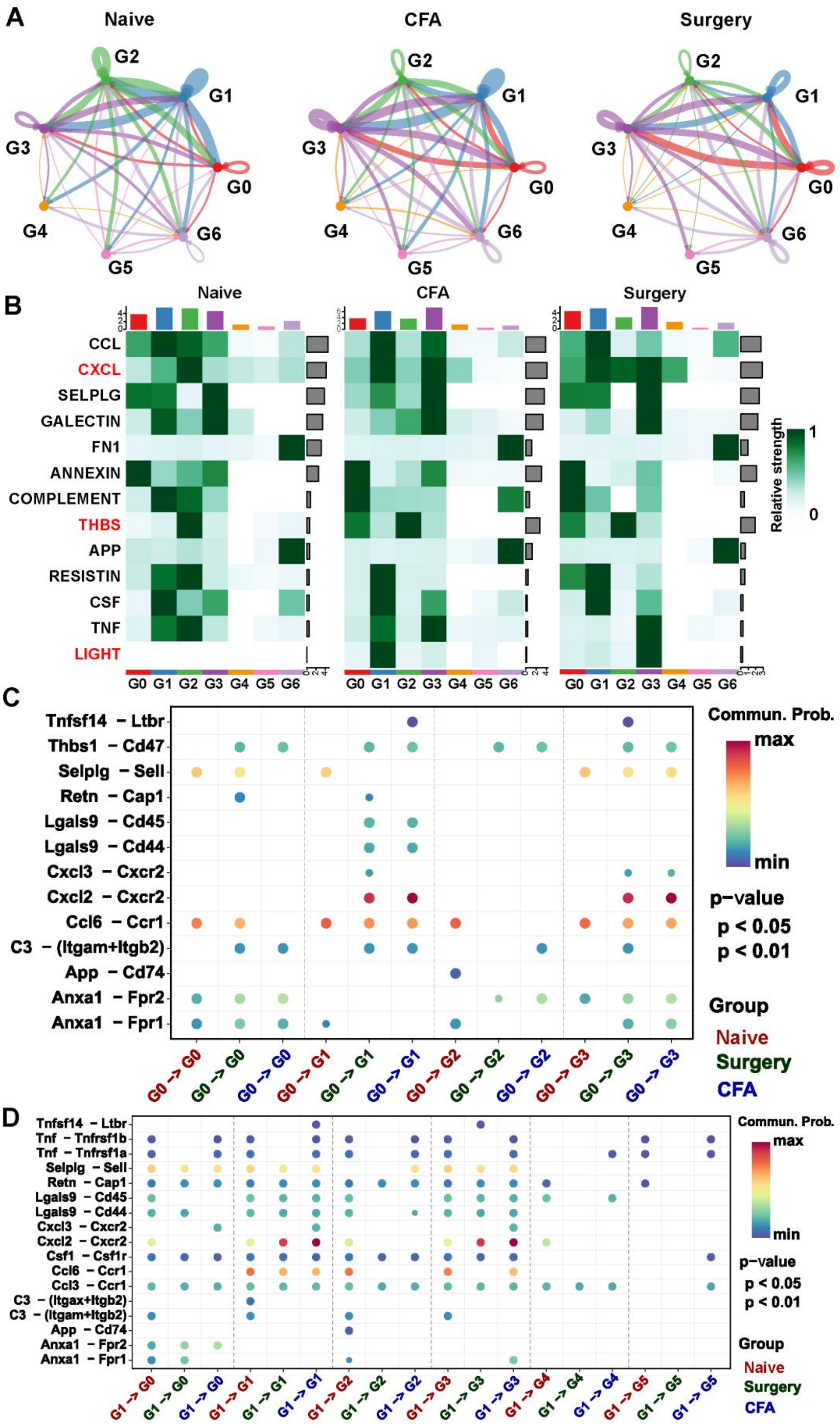

Supplementary Figure 6. The role of neutrophils in intercellular communication. (A) Network visualization of the communication between neutrophil subsets in the three models analyzed via the KEGG dataset. The line width indicates the communication

strength; the arrow direction signifies the signal flow from sender to receiver. (B) Heatmaps of signaling pathways for six neutrophil subsets across the three models. The top color bar represents the intensity score of overall signaling for the subsets, whereas the gray bar denotes intensity across all subsets. (C-D) Dot plots illustrating differences in ligand–receptor interaction strength, with G0 and G1 acting as signal transmitters. The dot size signifies the P value magnitude, and the color indicates the interaction strength.

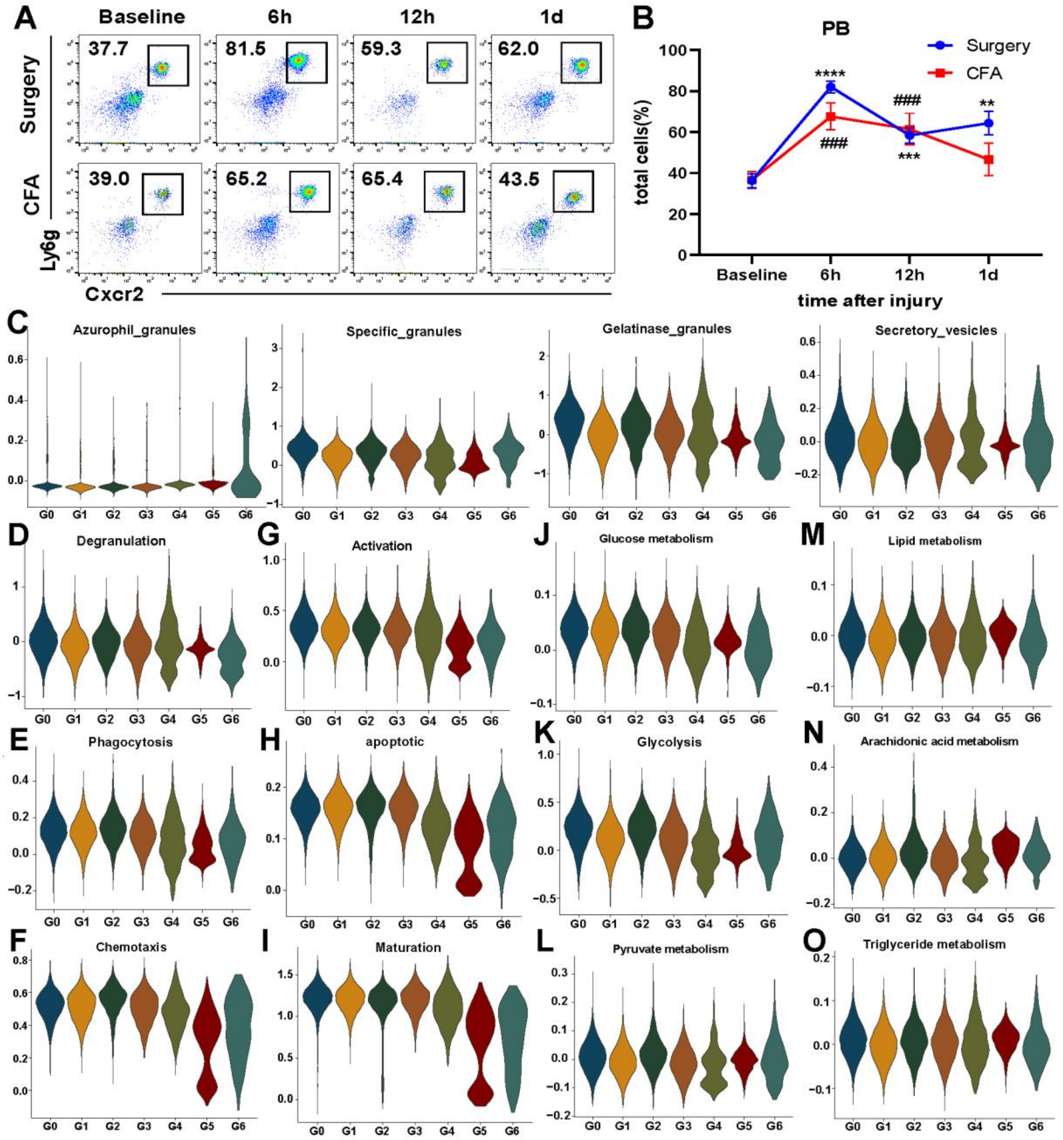

Supplementary Figure 7. (A-B) Flow cytometry (FCM) detection of early CXCR2 receptor expression in the peripheral blood of surgical and CFA-treated mice. The data are presented as the means ± SD. Statistical significance compared with baseline:

surgery ****P < 0.0001, ***P < 0.001, **P < 0.01; CFA ###P < 0.001, one-way repeated-measures ANOVA; n = 5 per group. (C) The functional scores of four-level particles in each subgroup of neutrophil. The four granule types are, in sequence: azurophil granules, specific granules, gelatinous granules and secretory vesicles. (D-I) Functional scores of each subgroup of neutrophil. Neutrophil degranulation (D), phagocytosis (E), chemotaxis (F), activation (G), apoptosis (H) and maturation (I). (J-O) Changes in neutrophil metabolism in subpopulations. (J-L) Changes in glycometabolism-related functions in subpopulations of neutrophils. Glucose metabolism (J), glycolysis (K) and pyruvate metabolism (L). (M-O) Scores of lipid metabolism related functions of each subgroup. Lipid metabolism (M), arachidonic acid metabolism (N) and triglyceride metabolism (O).

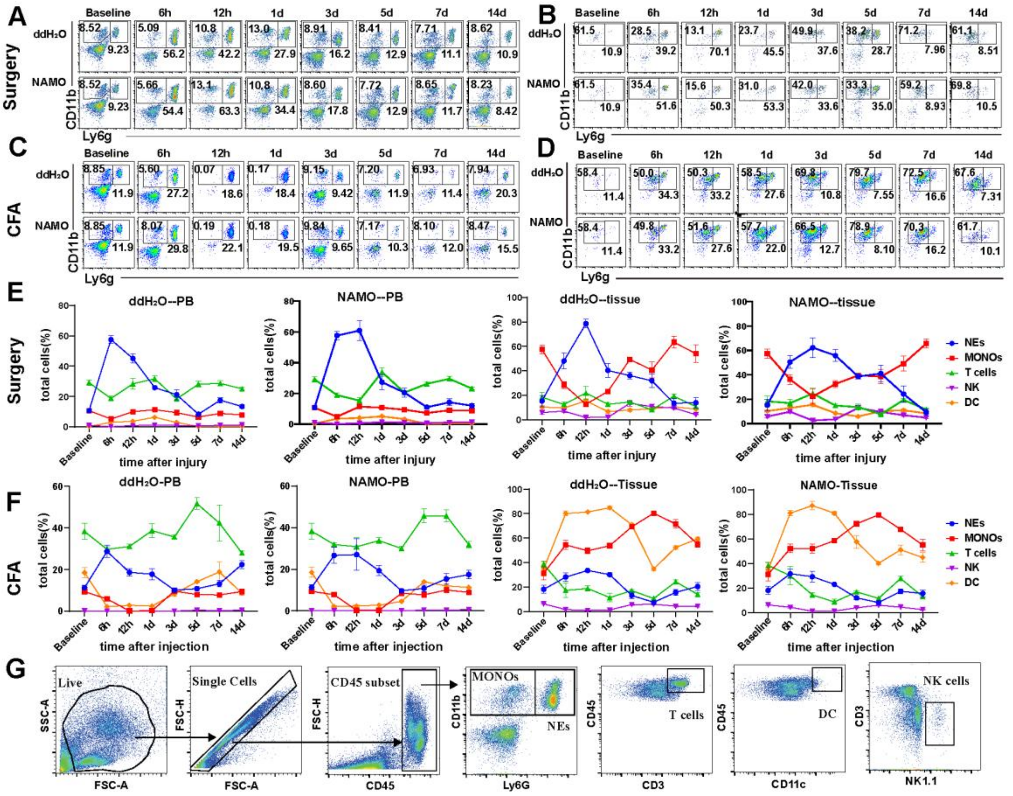

Supplementary Figure 8. FCM detection of immune cell changes. (A-B) FCM scatter plots illustrating temporal changes in neutrophils and monocytes in the peripheral blood and plantar tissues of surgical mice. (C-D) FCM scatter plots showing temporal changes in neutrophils and monocytes in the peripheral blood and

tissue of CFA-treated mice. (E-F) Line graphs of FCM results for other cell subpopulations in both surgical and CFA-treated mice over time. The data are presented as the means ± SEMs (n = 5–6 mice per group) from two independent experiments. (G) FCM detection protocol for the described experiment.
